# Synthetic prions with novel strain-specified properties

**DOI:** 10.1371/journal.ppat.1005354

**Published:** 2015-12-31

**Authors:** Fabio Moda, Thanh-Nhat T. Le, Suzana Aulić, Edoardo Bistaffa, Ilaria Campagnani, Tommaso Virgilio, Antonio Indaco, Luisa Palamara, Olivier Andréoletti, Fabrizio Tagliavini, Giuseppe Legname

**Affiliations:** 1 Unit of Neuropathology and Neurology 5, IRCCS Foundation Carlo Besta Neurological Institute, Milano, Italy; 2 Laboratory of Prion Biology, Department of Neuroscience, Scuola Internazionale Superiore di Studi Avanzati (SISSA), Trieste, Ital,y; 3 UMR INRA-ENVT, Physiopathologie Infectieuse et Parasitaire des Ruminants, Ecole Nationale Vétérinaire de Toulouse, Toulouse, France; 4 ELETTRA Laboratory, Sincrotrone Trieste S.C.p.A, Basovizza, Trieste, Italy; University of Alberta, CANADA

## Abstract

Prions are infectious proteins that possess multiple self-propagating structures. The information for strains and structural specific barriers appears to be contained exclusively in the folding of the pathological isoform, PrP^Sc^. Many recent studies determined that *de novo* prion strains could be generated *in vitro* from the structural conversion of recombinant (rec) prion protein (PrP) into amyloidal structures. Our aim was to elucidate the conformational diversity of pathological recPrP amyloids and their biological activities, as well as to gain novel insights in characterizing molecular events involved in mammalian prion conversion and propagation. To this end we generated infectious materials that possess different conformational structures. Our methodology for the prion conversion of recPrP required only purified rec full-length mouse (Mo) PrP and common chemicals. Neither infected brain extracts nor amplified PrP^Sc^ were used. Following two different *in vitro* protocols recMoPrP converted to amyloid fibrils without any seeding factor. Mouse hypothalamic GT1 and neuroblastoma N2a cell lines were infected with these amyloid preparations as fast screening methodology to characterize the infectious materials. Remarkably, a large number of amyloid preparations were able to induce the conformational change of endogenous PrP^C^ to harbor several distinctive proteinase-resistant PrP forms. One such preparation was characterized *in vivo* habouring a synthetic prion with novel strain specified neuropathological and biochemical properties.

## Author Summary

Prions are infectious proteins capable of acquiring multiple self-propagating structures. The information for strains and structural specific barriers appears to be contained exclusively in the folding of the pathological isoform, designated as PrP^Sc^. During propagation, disease-associated conformer PrP^Sc^ coerces the physiological form, denoted as PrP^C^, to adopt the pathological isoform conformation. We describe here the generation of an array of infectious materials with different structural, morphological, biochemical and cell biological characteristics. After producing purified recombinant prion protein of the wild-type mouse full-length sequence in *Escherichia coli*, we polymerized the protein into various amyloid fibril conformations based on different amyloid preparations. We also applied a build-in methodology for screening amyloid preparations and generate infectious materials using an amyloid-infected cell culture assay. Some of the amyloid fibrils preparations were able to efficiently amplify in PMCA (Protein Misfolding Cyclic Amplification), and to induce endogenous PrP^C^ to convert into PrP^Sc^ in both murine hypothalamic GT1 and neuroblastoma N2a cell lines. One such protocol lead to the generation of a novel synthetic prion strain in mice.

## Introduction

Prion diseases or transmissible spongiform encephalopathies are fatal neurodegenerative disorders of humans and animals. During the course of these maladies the cellular prion protein (PrP^C^) converts into an abnormally folded isoform, PrP^Sc^, which accumulates in the central nervous system (CNS), ultimately leading to the host death [1, 2]. The key molecular event of these disorders is therefore the conformational change of PrP^C^ from its physiological form into the pathological structural isoform noted as prion or PrP^Sc^. The physiological PrP^C^ is a glycosylphosphatidylinositol (GPI)-anchored protein present in all cell types. Unlike PrP^Sc^, PrP^C^ has a high content of α-helix in its secondary structure. Differences in structural conformation lead to different biological characteristics: while PrP^C^ is soluble in detergents and sensitive to proteinase K (PK) digestion, PrP^Sc^ is partially PK-resistant and insoluble in non-ionic detergents [3]. Nevertheless, a sizeable fraction of PrP^Sc^ is sensitive to PK digestion [4] and is dispersed with detergent and/or sonication [5]. Moreover, Colby *et al*. reported that protease-sensitive synthetic prions could be generated *in vitro* during polymerization of recombinant PrP (recPrP) into amyloid fibers [6]. Recently, PK-sensitive and PK-resistant PrP^Sc^ were shown to share a common structure and phenotype despite the differences in resistance to PK-digestion, sediment and distribution of multimers [7, 8].

For most proteins, if not all, the same amino acid sequence can encipher numerous and different amyloid states [9, 10]. The ability of PrP to acquire multiple self-propagating structures can thus explain the formation of multiple prion strains within the same host [11]. The information for prions is enciphered in these structures by a distinct conformation of the pathological isoform [12–14].

Synthetic prions were produced via *in vitro* induction of misfolding and aggregation of bacterially expressed recPrP [15]. This work clearly indicates that PrP^Sc^ is the sole component of the infectious agent, which propagates by converting PrP into various misfolded forms [14–16]. These first synthetic prions were produced injecting amyloid fibrils of recombinant mouse PrP residues 89–230 (recMoPrP(89–230)) into transgenic (Tg) mice carrying the homologous sequence. This endeavor opened new avenues in the structural characterization of infectious prions [15]. An array of recPrP amyloids with varying conformation stability was produced, showing a direct relationship between stability and incubation times of prion strains, at least in mice. The conformational stabilities of the new synthetic prion strains and their incubation periods seem to be dictated by the properties of the amyloid preparations from which they were generated [16]. Although lacking both glycolsylation and the GPI anchor, secondary and tertiary structures of refolded recPrP appear to be identical to those of brain-derived PrP^C^ [17, 18]. Remarkably, different amyloid preparations generated by recPrP can produce new prion strains with novel neuropathological and biochemical features when injected in mice [14–16]. This approach provided a useful tool to further investigate the functional/structural relationships of mammalian prions. In the last few years, different protocols have been established in which recPrP was successfully converted into PrP^Sc^ by means of Protein Misfolding Cyclic Amplification (PMCA) [19, 20]. This technique consists of cycles of sonication and incubation which uses normal brain homogenate as source of PrP^C^ [21]. The crystal structure of human recPrP has revealed a possible mechanism for oligomerization in which the three-dimensional swapping of the C-terminus helix 3 and the re-arrangement of the disulfide bond result in the formation of a dimer [22, 23]. These data have suggested a possible role for a sulfhydryl-disulfide exchange reaction during the conversion of PrP^C^ to PrP^Sc^. Moreover, this mechanism has been recapitulated *in vitro* by seeded conversion of hamster recPrP(90–231) truncated form with an histidine tag (His-tag) to a disulfide-bonded oligomer by a reduction-oxidation (REDOX) process [24].

We therefore established two different experimental procedures for amyloid preparations: (i) REDOX process, and (ii) non-REDOX process, using full-length recMoPrP residue 23 to 231 (recMoPrP(23–231)). Our protocols for conversion of PrP required only purified full-length recMoPrP and common chemicals. We were then able to produce 34 different amyloids. Neither prion-infected brain extracts, nor amplified exogenous PrP^Sc^ were used. In both processes, recMoPrP(23–231) converted to conformational structures of fibrils without any seeded factor. The fibrils generated by the REDOX process most likely contained intermolecular disulfide bridge structures. In addition, the recMoPrP(23–231) amyloid preparations subjected to the REDOX and non-REDOX *in vitro* processes exhibited different morphological and biochemical prion characteristics. The ability of these amyloid preparations to behave as *bona-fide* prion were assessed *in vitro* either in cell cultures or by means of PMCA.

First, we employed an amyloid-infected cell culture assay for screening amyloid preparations, in order to evaluate putative infectious materials [25]. Based on this methodology, when infected into mouse hypothalamic (GT1) and mouse neuroblastoma (N2a) host cell lines [26], amyloid fibrils from different preparations induced the conversion of endogenous PrP^C^ to mildly PK-resistant PrP isoforms. The various amyloid preparations also caused PrP to accumulate and propagate in both membrane and cytosolic compartments of cultured neuronal cells. Two of the amyloid preparations that were shown to efficiently propagate in cell culture and produced under either REDOX or non-REDOX conditions, #28 and #4 respectively, were assessed by means of PMCA. To this aim we used the brain of wild-type mice (CD1) as substrate for amplification. Finally, these two preparations were intracerebrally injected (i.c.) into CD1 mice to assess their ability to induce prion pathology *in vivo*. While preparation #28 did not produce any evident prion pathology, isolate #4 was able to replicate and induce clinical disease in wild-type CD1 mice harbouring distinctive neuropathological and biochemical features.

## Results

### Generation of different recPrP amyloids

To generate recPrP amyloids with different conformations, we systematically altered the conditions for their formation, including denaturant concentrations, pH and buffer composition (S1 and S2 Tables). The mechanism of amyloid formation determines significantly the amyloid structure of recPrP. The protein has been known to convert to different types of amyloid and some assemblies may be induced with the formation of intermolecular disulfide bonds leading to domain-swapping [27, 28]. These alternative structures showed the coexistence of different molecular forms of PrP with the capacity of self-propagating prions [24]. In our experiments, we started from different states of recMoPrP(23–231) using either REDOX or non-REDOX processes. This resulted in different amyloid preparations in which the monomeric recMoPrP(23–231) followed different pathways for more stable free energy states. We induced the REDOX process by reducing disulfide bonds of recMoPrP(23–231) at high concentration of reducing agent in the presence of 6M guanidine hydrochloride (Gdn-HCl), with or without the addition of sodium chloride (NaCl). The concentration of all these components and of the protein was subsequently decreased by means of direct dilution to reach the final concentrations for fibrillization reactions (S1 Fig). This process differs from the earlier protocol reported to convert PrP *in vitro* [24], although sharing the same mechanism [27, 28]. In order to study the converted ability and folding behaviors of recMoPrP(23–231), we analyzed the kinetics of purified recMoPrP(23–231) conversion into β-sheet-rich forms under different defined biophysical and biochemical conditions (S1 and S2 Tables).

### Morphological and biochemical characterization of recPrP amyloids

**Fig 1:**
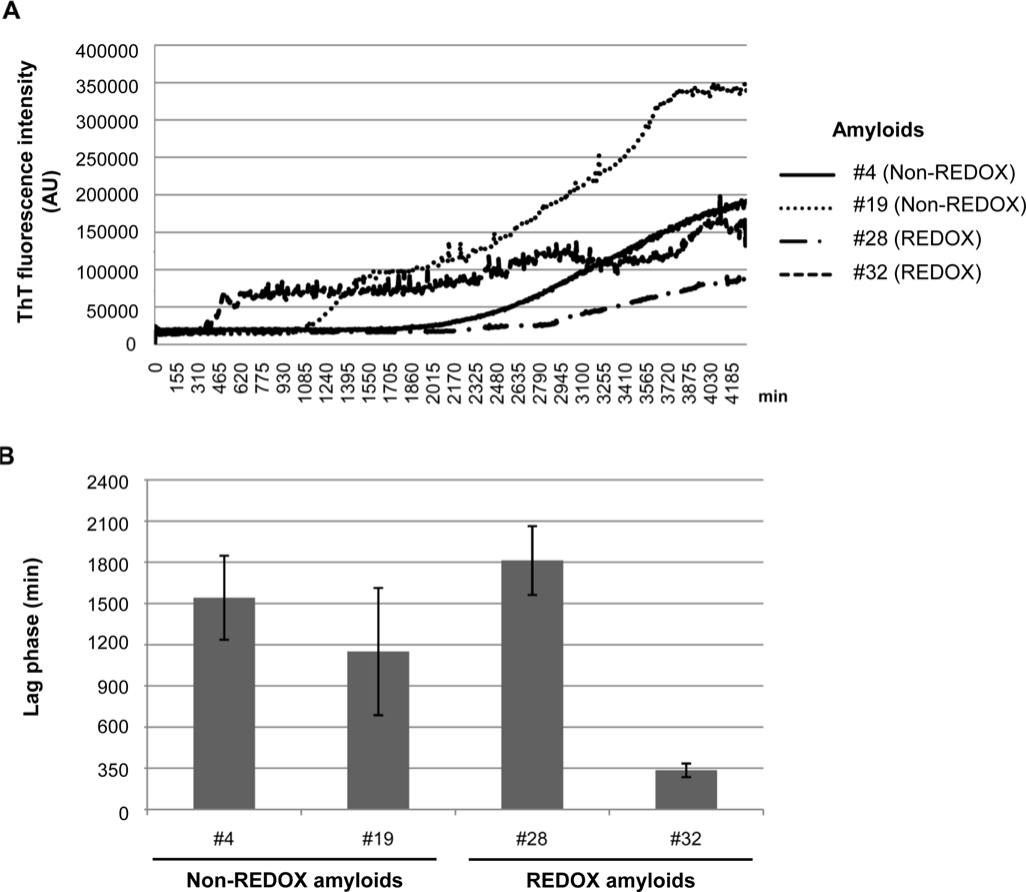
Aggregation assay of recMoPrP(23–231). Recombinant mouse PrP residues 23–231 (recMoPrP(23–231)) was converted *in vitro* into different amyloid forms (amyloids #4, #19, #28, #32). The amyloid preparations shown (**A**) exhibited different kinetics for the formation of recMoPrP(23–231) aggregates. Lag phase distribution of amyloid preparations. Bars indicate standard deviation (**B**).

To monitor the amyloid formations, we used a thioflavin-T (ThT)-binding assay [29, 30]. Kinetic curves presented a sigmoidal shape, typically denoting an increased content of β-sheet structures, as well as enhanced aggregation of monomeric recPrP (Fig 1A). These sigmoidal curves highlighted a lag phase followed by rapid accumulation of fibrils (Figs 1B and S2). The quantitative analysis of the lag phase was carried out by estimating the increase in ThT fluorescence intensity [31]. The end of the lag phase coincides with the point when ThT fluorescence intensity started to increase. The maximum ThT fluorescence intensities exhibited a wide range between the two amyloid preparation processes. In the non-REDOX process, the maximum intensities are higher than those of the REDOX process (Fig 1A). At neutral pH, the lag phases for aggregation processes of recMoPrP(23–231) are shorter in comparison with acidic pH 5 (S2 Fig). The shortest lag phase and most rapid production of amyloid were observed at pH 7.4 (S2 Fig) (p<0.05, n = 12). Interestingly, the fibrillization showed that lag phases vary in the presence of 2M Gdn-HCl between different conditions and also under the same conditions, which were indicated with high error bar values (S2 Fig). These data suggest that at different concentrations of Gdn-HCl, monomeric recMoPrP(23–231) explores many states of accessible free energies [32]. At denaturant concentration less than 1M, production of amyloids was observed only at pH 7.5 in the REDOX process, whereas no amyloid form was found at pH 5.0.

**Fig 2:**
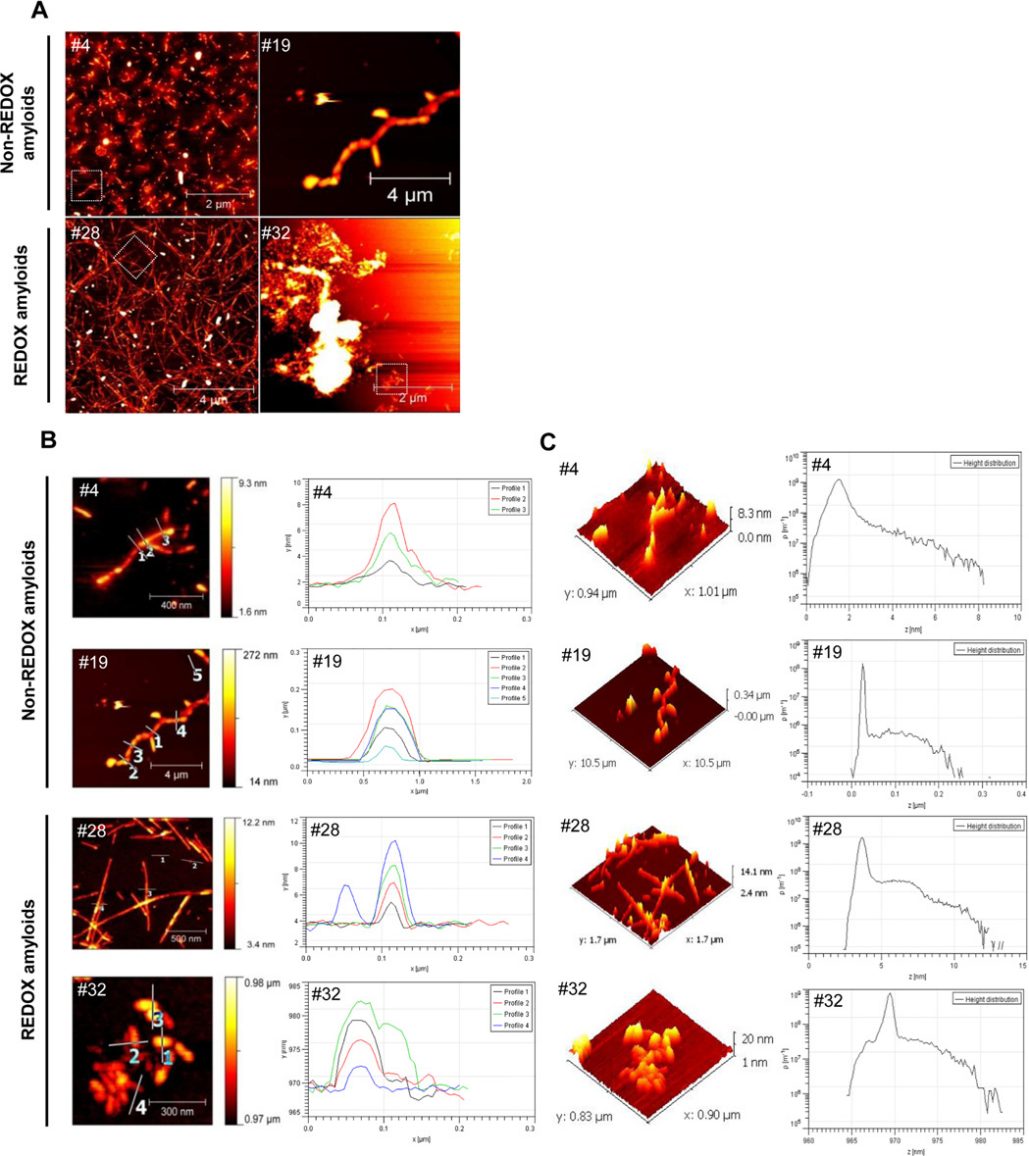
AFM characterization of amyloid fibrils. Atomic Force Microscopy (AFM) imaging analysis was performed at the end of the fibrillization reactions after 72 hours (amyloids #4, #19, #28, #32). AFM scan topographical images of prion protein (PrP) deposited on mica surface, large-scale images (**A**). AFM height profiles along the numbered lines in topographical images. The profile reflects the lines as numbered in the images. Higher resolution scan images belonging to the area are marked by a white dashed square in part A (**B**). Three-dimensional representation of AFM topography images and height distribution data obtained from the AFM images in part B (**C**).

Differences in kinetic traces and maximum intensity values (Figs 1 and S2) were also highlighted in terms of morphology, as revealed by atomic force microscopy (AFM) (Figs 2 and S3A).

To gain further insights into the aggregated morphology of the end products, we studied the aggregated topology of all recMoPrP(23–231) amyloid preparations by AFM. The recMoPrP(23–231) aggregation products were imaged at two end point times of fibrillization, i.e. 55 hours and 72 hours (Figs 2 and S3A). AFM scans of mica surfaces treated with different amyloid preparations showed that recMoPrP(23–231) aggregated after 72 hours and clearly revealed marked morphological differences. The morphology-dependence of the fibrillizations was observed at different concentrations of denaturant (S3B Fig). For example, at 4M Gdn-HCl and 4M Gdn-HCl with NaCl in both REDOX and non-REDOX processes, the AFM analyses displayed a relatively homogenous population of spherical particles (β-oligomers) [33] and very short fibrils (approximately less than 0.5 μm) (Figs 2 and S3A). In both processes, at 2M Gdn-HCl, 2M Gdn-HCl in the presence of NaCl and 3M Gdn-HCl alone, recMoPrP(23–231) was converted to amyloid forms. These were visualized by AFM as fibrils, ranging from very short to longer and more mature fibrils. The major fibrillar subtypes were straight, slightly curvy ribbons or rod-shaped fibrils (Figs 2 and S3A). These results are all in agreement with earlier studies [34]. In order to ascertain whether some preparations were true amyloids, further experiments were carried out to test their capability of seeding amyloid formation.

In our amyloid morphology analyses, different amyloid preparations of recMoPrP(23–231) showed aggregate clusters of different heights (Tables 1 and S3 and Figs 2B and S3). 3-D views and height distribution (Fig 2C) revealed structures with different topologies from different amyloid preparations.

**Table 1:** Different height of clusters of recMoPrP (23–231) aggregates.

Amyloid preparation (#)	[Gdn-HCl] (M)	Buffer	pH	Height (nm)	Top height distribution (nm)
**4**	4	50mM Acetate	5.0	1.8–2.2	1.8
**19**	2	PBS	7.4	25–50	25
**28**	3	50mM Acetate	5.0	1.2–2.5	2.5
**32**	1	PBS	7.4	2.5–4.5	4.5

The analyses of both AFM and kinetics data showed that different amyloid preparations in which pH, denaturing conditions, ionic strength and REDOX processes were defined, reflect differences in aggregation pathways and morphology of recMoPrP(23–231) end products.

**Fig 3:**
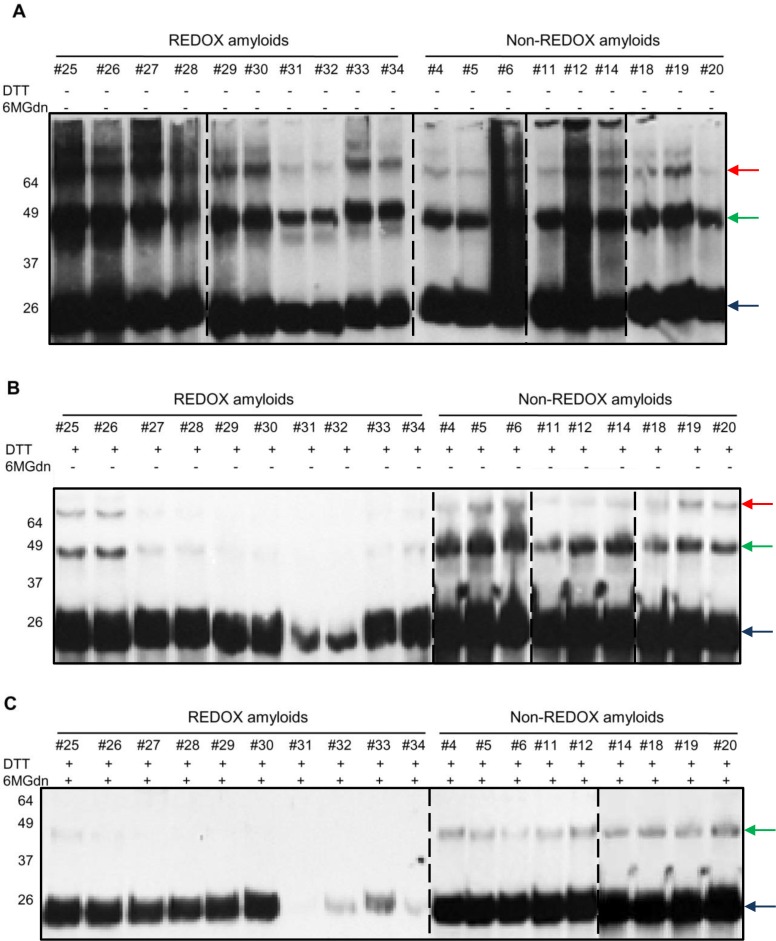
Western blot analysis of amyloid fibrils performed under reducing and non-reducing SDS-PAGE. Monomeric (indicated by blue arrow) recMoPrP(23–231) was converted into amyloid forms by intermolecular disulfide linkage following the REDOX process. Western blotting of non-reducing Sodium Dodecyl Sulphate—PolyAcrylamide Gel Electrophoresis (SDS-PAGE) showing the conversion of recMoPrP(23–231) to amyloids is indicated by dimer (green arrow), trimer (red arrow), and more complex structures in both processes (A). Western blotting of reducing SDS-PAGE after treatment of amyloid with Dithiothreitol reducing agent (DTT) shows the decrease in signals of dimer, trimer and more complicated structures in all lanes of amyloid samples from REDOX-process (B). Western blotting of reducing SDS-PAGE of amyloid after a 3-day treatment with denaturant (6M Gdn-HCl), and subsequently with reducing agent DTT shows only monomeric recMoPrP(23–231) bands and the disappearance of more complicated structures in all lanes of amyloid samples in REDOX process (C). Western blots were performed using Fab D18 monoclonal antibody (1μg/mL). Blots were developed with the enhanced chemiluminescent system (ECL, Amersham Biosciences) and visualized on Hyperfilm (Amersham Biosciences)

**Fig 4:**
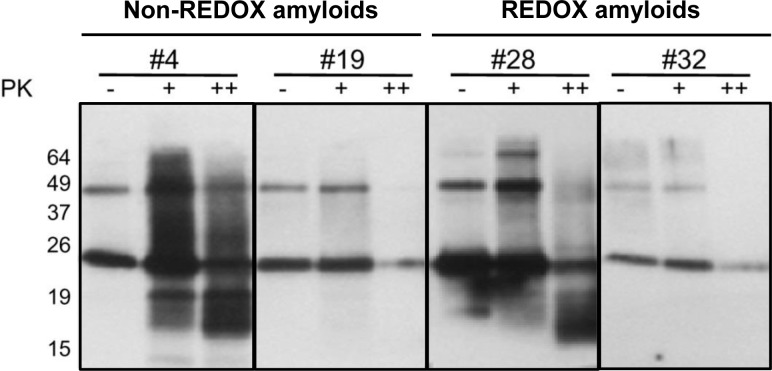
PK digestion assay of amyloid #4, #19, #28 and #32. Western blotting of PK digestion assay (amyloids #4, #19, #28, #32) showed partial protease K (PK) resistance of recMoPrP(23–231) (amyloids #4 and #28). RecMoPrP(23–231) amyloids (PK- lanes) were digested with PK at ratio 1:10 (w/w) (PK+ lanes) and 1:1 (w/w) (PK++ lanes). Western blots were performed using Fab D18 monoclonal antibody (1μg/mL). Blots were developed with the enhanced chemiluminescent system (ECL, Amersham Biosciences) and visualized on Hyperfilm (Amersham Biosciences)

As spontaneous air re-oxidation occurs in the REDOX amyloid preparation process, the disulfide bonds may rearrange, changing from monomeric intramolecular PrP form to intermolecular multimeric PrP. In order to prove that the converted amyloid forms under different amyloid preparations from the REDOX process were oligomerized through intermolecular disulfide bonds, we checked for the presence of these structures. In comparison with non-reducing experiments, we used a high concentration of reducing agent and reduced electrophoresis. Western blottings showed that converted amyloid forms from both processes had apparent monomeric, dimeric molecular masses and higher, more complex multimeric forms under non-reducing treatments (Fig 3A). After treatment with the reducing agents to disrupt all disulfide bridges, the REDOX-converted PrP forms showed a significant decrease in dimeric and trimeric structures (Fig 3B, REDOX amyloids). In amyloid preparations established following the REDOX process—which includes the swapping of domains and the rearrangement of intra and intermolecular disulfide bridges in PrP molecules—the conversions require both non-covalent and covalent bonds to break up. Moreover, induced reduction occurs optimally in denaturing condition. Therefore, after incubating the REDOX-converted forms with high concentration of denaturant (Gdn-HCl 6M) for three days and subsequently treating them with reducing agent, the amyloid forms were disassembled. This was indicated by the disappearance of PrP complexes with structures other than the monomeric form (Fig 3C, REDOX amyloids). In contrast, recMoPrP(23–231) converted into the amyloid forms in the non-REDOX process—in which the oxidized PrP was diluted to reach the final concentrations in fibrillization buffer—showed greater stability of dimeric and higher structures after treatment with reducing agent (Fig 3B and 3C, non-REDOX amyloids). Besides the differences in morphology and kinetics formation, our intermolecular disulfide bridge tests showed that REDOX amyloids are most likely to contain intermolecular disulfide bridge conformation structures [24, 27, 28]. So far, PK-resistance has been used to distinguish PrP^C^ from PrP^Sc^, although many studies showed the existence of PK-sensitive PrP^Sc^. We treated amyloid fibrils at PK/recMoPrP(23–231) ratios of 1:10 and under standard conditions used for detecting PrP^Sc^ in brain homogenates (20 μg/mL PK, at 37°C for 1 hour) at the ratio of 1:1. This process aimed at determining whether: (i) our abnormal isoforms of PrP generated *in vitro* are resistant to PK digestion, and (ii) they have a PK-resistant core resembling that of PrP^Sc^ subjected to PK treatment. Both the β-oligomers and the amyloid fibrils in some amyloid preparations showed PK-resistance. Interestingly, in both processes, most amyloid preparations in Phosphate Buffer Saline (PBS) pH 7.4 appeared more sensitive to PK digestion than those in acidic conditions, although digestion patterns were undistinguishable (Figs 4 and S4). At ratio 1:10, the PK-resistant bands of non-REDOX amyloid digestion displayed several resistant fragments with molecular masses in the range of ~17–19 kDa. Only in the case of amyloid preparations in acidic pH, molecular masses were less than 15 kDa. By contrast, REDOX amyloid fibrils exhibited PK-resistant bands of molecular masses in the range of ~17–19 kDa (Figs 4 and S4). In particular, after treatment with PK at ratio 1:1, amyloid fibril #28 showed bands with molecular mass of ~16 kDa (Fig 4). Notably, amyloid preparations subjected to the REDOX process in neutral buffers (S4 Fig) showed most PK-sensitive structures. Upon treatment with high concentration of PK, nearly all of these amyloid preparations were almost entirely digested, showing only trace amounts of similar kDa bands. The biochemical analyses of our amyloid preparations showed that in different biochemical and biophysical environments, recMoPrP(23–231) converted to distinct amyloidal forms. These different structures exhibited distinct stability in the presence of high concentration of denaturant (Fig 3C), PK treatment (Figs 4 and S4) and molecular masses patterning of PK-resistant cores. Kinetic studies of fibrillization (S2 Fig) showed that at neutral pH, the lag phase of recMoPrP(23–231) was short, and the protein was more prone to generate amyloids different from those formed at different pH. Interestingly, the stability of amyloid forms under neutral pH condition was lower.

The molecular basis of prion infectivity is the ability of PrP^Sc^ to efficiently induce the conversion of PrP^C^ into PrP^Sc^. This process follows the seeding-nucleation model, with infectious PrP^Sc^ acting as a seed to capture PrP^C^ into a prion polymer [35].

### Seeding ability in cell cultures

**Fig 5:**
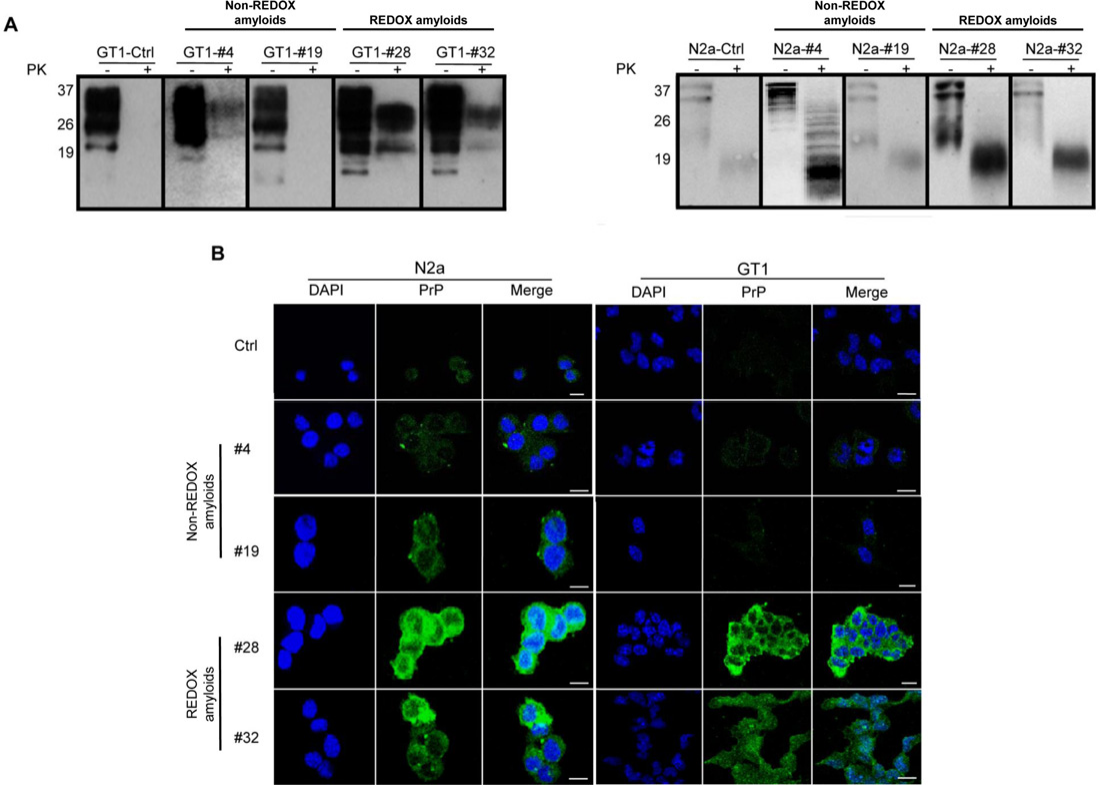
Seeding assay of N2a and GT1 cell cultures with synthetic amyloid fibrils. Seeding of recMoPrP(23–231) amyloid preparations induced the conversion of endogenous PrP^C^ to mildly PK resistant forms (A) and accumulation (B) in mouse neuroblastoma N2a and mouse hypothalamic GT1 amyloid-infected cell lines analyzed six passages after the infection (P6). Western blotting shows the partial protease K (PK) resistance of N2a and GT1 amyloid fibril-infected cell lysates. Fibril-infected cell lysates (PK-lanes) were digested with PK at ratio 1:500 (w/w) (PK+ lanes). Western blots were performed using Fab D18 monoclonal antibody (1μg/mL) for GT1 infected cells and Clone-P (1μg/mL) for N2a infected cells. Blots were developed with the enhanced chemiluminescent system (ECL, Amersham Biosciences) and visualized on Hyperfilm (Amersham Biosciences) (A). Immunofluorescence imaging shows the accumulations of PrP in N2a and GT1 amyloid fibril-infected cell lines. The deposition and level of PrP (green) in amyloid fibril-infected cell lines after six passages were detected by Fab D18 monoclonal antibody (10 μg/mL final concentration). The nuclei (blue) were stained with DAPI. Scale bar is 20μm (B).

**Fig 6:**
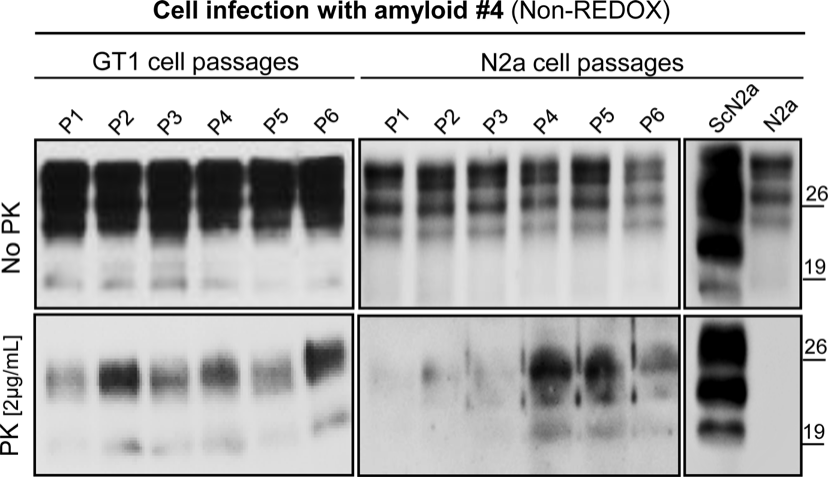
PK digestion assay of GT1 and N2a cells collected at different passages after infection with amyloid fibrils. Western blotting of GT1 and N2a cell lines infected with PrP amyloid #4 was observed throughout, from first passage (P1) to sixth passage (P6) and after treatment with proteinase K at ratio 1:500 (w/w). Western blot was performed using Fab D18 monoclonal antibody (1μg/mL). Blots were developed with the enhanced chemiluminescent system (ECL, Amersham Biosciences) and visualized on Hyperfilm (Amersham Biosciences).

Using cell-cultured models for the screening of several amyloid preparations, we seeded our amyloid fibrils to cultured mouse hypothalamic GT1, mouse neuroblastoma N2a cells and mouse hippocampal knockout PrP HpL3-4 cells [36]. After six cell passages, mildly PK-resistant PrP (Figs 5A and S5) and aggregated forms of PrP were found in amyloid fibril-infected GT1 as well as in N2a cells (Figs 5B, S6 and S7), whereas HpL3-4 knockout PrP cells did not harbor any detectable aggregated PrP. Throughout serial passages, endogenous PrP^C^ was induced to change its conformation to mildly PK-resistant PrP forms, which were maintained after passages (Fig 6).

Some preparations induced aggregation only in one cell line (S5, S6 and S7 Figs). Notably, different batches of the same amyloid, prepared at different times, were characterized by similar PK resistance and infectious properties, either in cell lines and by means of PMCA (S12 Fig). Cell lines used in this work derived from different sources, which may account for the diverse susceptibility to various conformations of putatively infectious PrP. Interestingly, incubation with some amyloid preparations did not promote PK-resistant PrP formation in either GT1 or N2a cells (Figs 5A and S5).

The immunofluorescence analyses of amyloid fibril-infected cell lines (Fig 5B) showed different staining and stronger PrP immunoreactivity detection than those of uninfected cells in both N2a (S6 Fig) and GT1 (S7 Fig) cell lines. Some cells showed accumulation, indicated by punctates and clusters of immune signal. Detected PrP accumulation in amyloid fibril-infected cells was found at the cell membrane and in the cytosolic compartments.

These data suggested that amyloid preparations, in both REDOX and non-REDOX conditions, can act as seeds, like natural prions do, in propagating within cultured neuronal cells, leading to accumulation and promotion of PK-resistant PrP forms from endogenous PrP.

In several recent independent studies on different proteins, both synthetic and natural amyloid forms have been shown to induce apoptosis in cell cultures [37–39] thus suggesting that these amyloids might be toxic. To check for possible toxic effects on our neuronal cell lines, we measured cell viability after treatment with amyloid preparations in a similar procedure with amyloid fibril-infected cell assay using MTT (3-(4,5-Dimethylthiazol-2-yl)-2,5-Diphenyltetrazolium Bromide) assay. Indeed, some amyloid preparations showed toxicity in cell culture while most did not (S8 Fig).

### PMCA propagation and infectivity in wild-type animals

**Fig 7:**
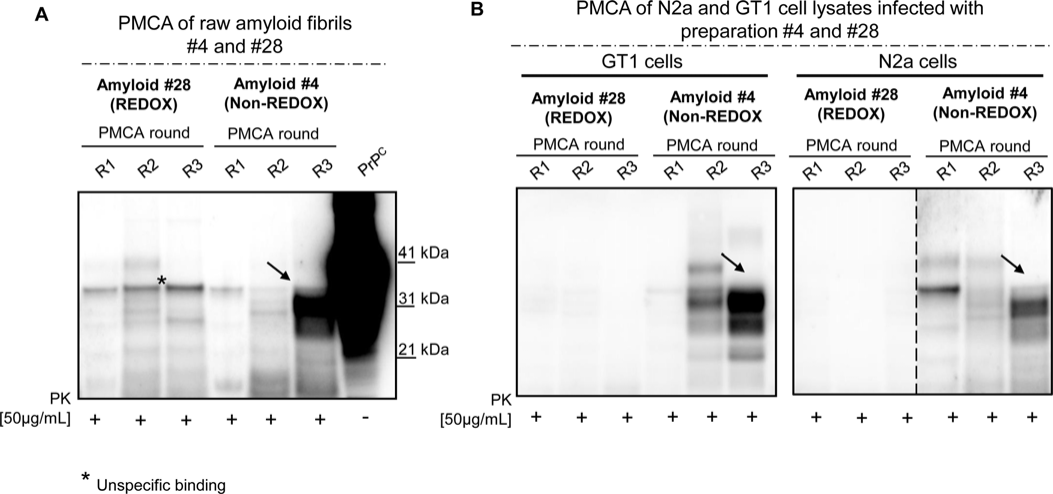
PMCA analysis of raw fibrils and cell lysates from infected N2a and GT1 cell lines. Seeding ability of amyloid #4 and #28 by means of PMCA using brain homogenates of CD1 mice as substrates for amplification (A). PMCA analysis of GT1 and N2a cell lysates (infected with preparations #4 and #28) and collected at passage six (P6) after the infection (B). Black arrow indicates PK-resistant PrP. Asterisk indicates unspecific binding. Western blots were performed using 6D11 monoclonal antibody to PrP (0.2 μg/mL, Covance). Blots were developed with the enhanced chemiluminescent system (ECL, Amersham Biosciences) and visualized using a G:BOX Chemi Syngene System.

**Fig 8:**
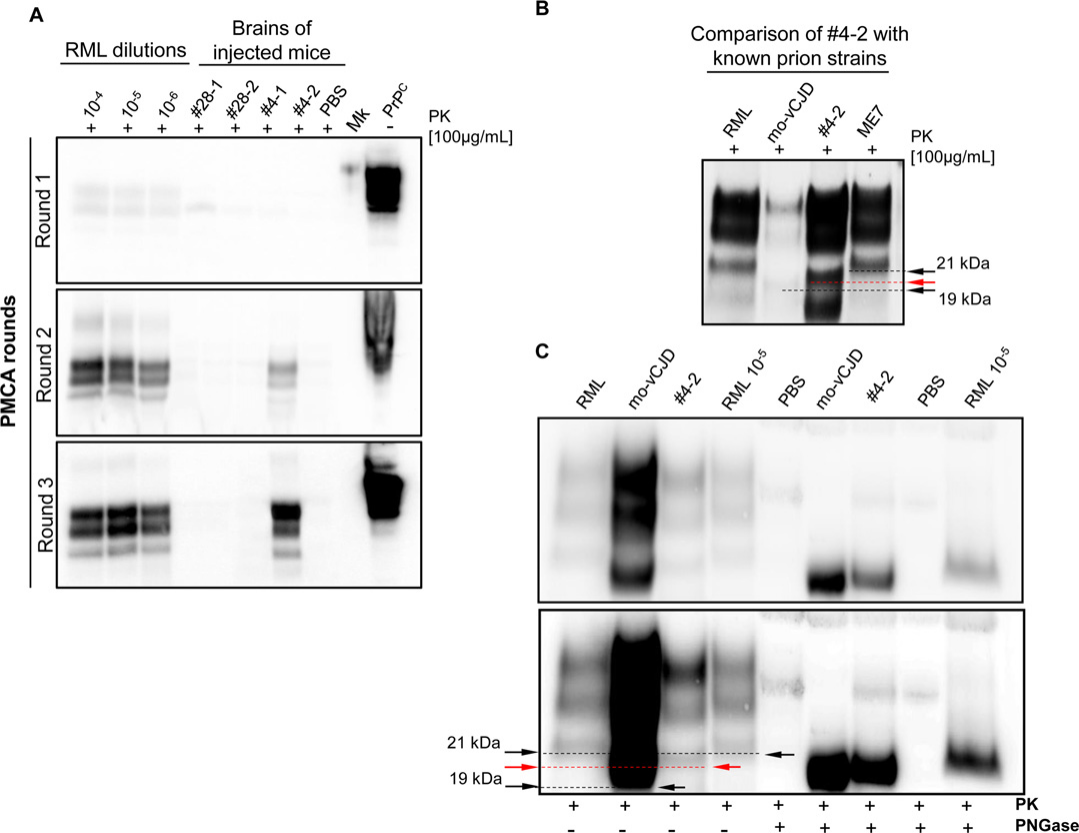
Amplification and characterization of a new prion isolate obtained from the brain of amyloid-infected animals. PMCA assessment using the brain homogenate of injected animals (with amyloid #4 or #28) as seed and the brain of wild type CD1 animals as substrate. Serial dilution of RML prion strain were used as internal control for PMCA efficiency (A). Western blot (B) and PNGase comparison (C) of amplified clone #4 (PMCA-#4) with known prion strains (RML, mouse adapted vCJD and ME7). Read arrows in B and C indicate the different electrophoretic mobility of the unglycosylated PrP band of PMCA-#4 (migrating at around 20kDa) compared to that of known prion strains migrating at 19 kDa or 21 kDa (black arrows). Western blots were performed using 6D11 monoclonal antibody to PrP (0.2 μg/mL, Covance). Blots were developed with the enhanced chemiluminescent system (ECL, Amersham Biosciences) and visualized using a G:BOX Chemi Syngene system.

Two of the amyloid preparations described in this manuscript, amyloid #4 and #28, were further analyzed by different techniques. Particularly, we assessed their ability to propagate *in vitro* by means of PMCA using the brain of CD1 mice as substrate for amplification. Only the preparation #4 showed a PK resistant PrP (PrP^Res^) signal after three rounds of PMCA while preparation #28 did not (Fig 7). Similarly, the cell lysates obtained from both N2a and GT1 cells infected with amyloid preparations #4 or #28 (collected at passage 6) were used as seed for PMCA reaction. The lysates of both cell lines infected with amyloid #4 were able to amplify in PMCA harboring a PK resistant signal after 3 rounds of amplification while preparation #28 was not able to amplify (Fig 7). Similar results were obtained in PMCA experiments performed using new batches of raw fibrils or lysates from cells infected with different preparations of the same amyloid (S12E Fig). Remarkably, the glycoform ratio of the amplified product derived from amyloid #4 (either from crude amyloid or from cell lysates) was always characterized by a prevalence of the diglycosylated band after PK digestion. The unglycosylated form of PrP^Res^ migrated at around 20 kDa (Fig 7). Finally, both amyloid preparations were intracerebrally inoculated in CD1 mice. A group of animals were injected with normal PBS as control. None of the animals injected with amyloid preparations showed any prion pathology and were culled at the end of their lifespan (720 days post injection). Immunohistochemical (S10A Fig) and biochemical (S10B Fig) analysis confirmed the lack of spongiosis, PrP^Res^ deposition and astroglial or microglial activation [40]. The presence of trace amounts of synthetic prion preparation in the brain of these animals was analyzed by means of PMCA. In accordance with the *in vitro* results, only the animals injected with preparation #4 showed the presence of a PrP^Res^ signal after 2 rounds of PMCA while we could not detect any signal when analyzing the brain of the animals injected with preparation #28 (Fig 8A). The PrP^Res^ amplified in preparation #4 (PMCA-#4) was biochemically analyzed and compared with known prion strains: (i) mouse adapted-variant CJD (vCJD), (ii) RML and (iii) ME7. We noticed that the unglycosylated band of PMCA-#4 migrated at around 20 kDa, in an intermediate position compared with the one of RML or ME7 and the one of mouse adapted-vCJD (Fig 8B). These results were confirmed with Peptide-N-Glycosidase (PNGase) experiment (Fig 8C). The higher molecular weight (20 kDa) of the unglycosylated band of PMCA-#4 compared to the one of the synthetic preparation (17–19 kDa) might be explain by the presence of the GPI anchor which is added *in vivo*. With this experiment we could also rule out the possibility that the signal obtained in PMCA-#4 could have derived from PMCA contamination with Bovine Spongiform Encephalopathy (BSE) or vCJD (known to have a prevalence of the diglycosylated form of PrP^Res^).

**Fig 9:**
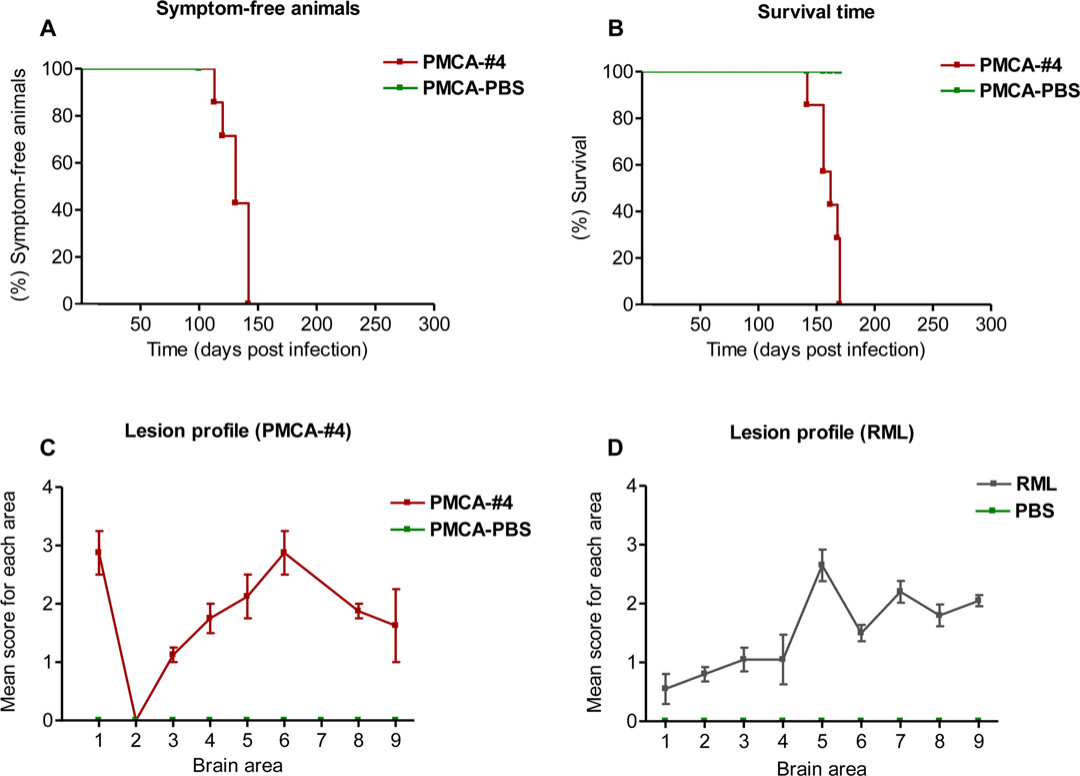
Incubation time, survival time and lesion profile analysis of PMCA-#4 infected animals. The animals injected with PMCA-#4 showed an incubation (A) and survival time (B) of 130 ± 4.4 and 160 ± 3.85 days (Mean ± Standard Error of the Mean, SEM) and results were analyzed with the Logrank test. Spongiform profiles were determined on Hematoxylin and Eosin (H&E)-stained sections, by scoring the vacuolar changes in nine standard gray matter areas: 1. Dorsal medulla; 2. Cerebellar cortex; 3. Superior culliculus; 4. Hypothalamus; 5. Thalamus; 6. Hippocampus; 7. Septum; 8. Retrosplenial and adjacent motor cortex; 9. Cingulated and adjacent motor cortex, as described by Fraser et al. [54]. Lesion profile was compared to that of RML (i.c.) infected animals. Bars in C and D indicate Standard Error of the Mean.

**Fig 10:**
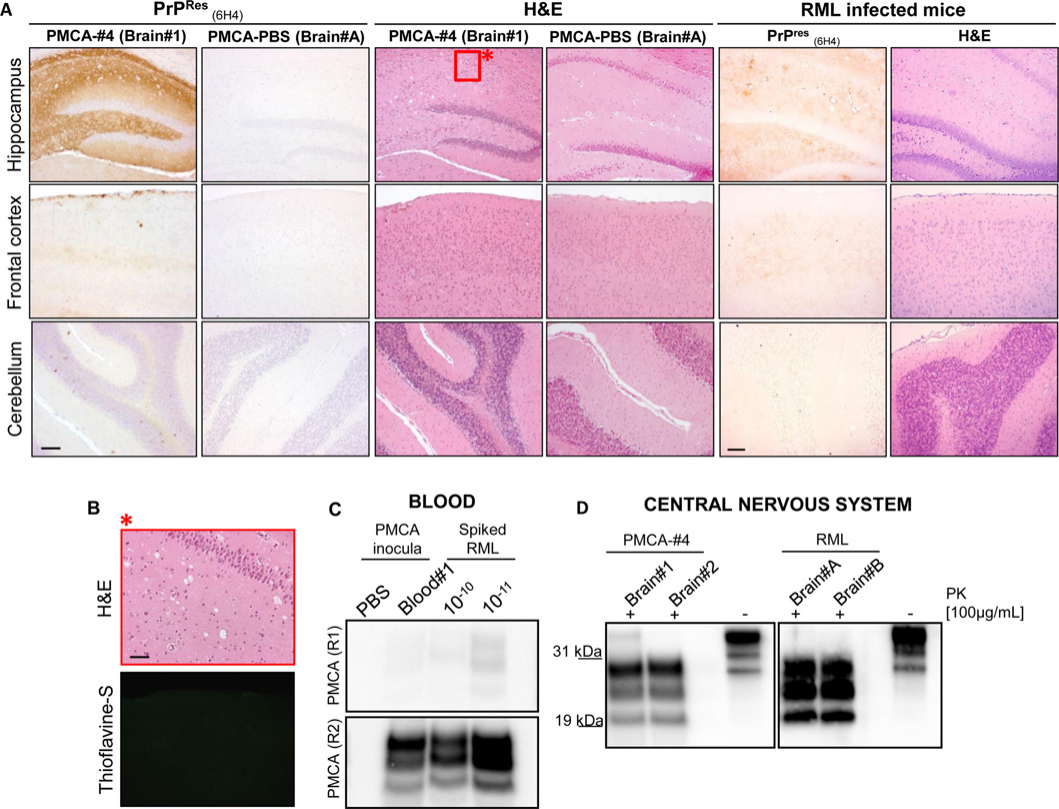
Detection of PMCA-#4 in brain and blood of infected animals and related immunohistochemical/biochemical characterization. Neuropathological analysis of PMCA-#4 and PMCA-PBS injected animals and comparison to that of RML infected mice. Animals injected with PMCA-#4 showed widespread deposition of PrP^Res^ in the hippocampus with focal plaque-like deposits found in the cerebral cortex. Cerebellum is completely spared by PrP^Res^ accumulation. RML injected animals showed the typical pattern of widespread, synaptic and diffuse PrP^Res^ accumulation in the whole brain with major involvement of thalamus and hippocampus. Spongiform changes were mainly found in the hippocampus of PMCA-#4 injected animals. Few vacuoles were detected in the cerebral cortex, while cerebellum did not show any vacuolation. RML injected animals showed severe vacuolation in the thalamus and hippocampus. Mild alterations were found in cerebellum and septum. Brain of PMCA-PBS injected animal was used as control (A). Higher magnification of the red square in panel A (see asterisk) and Thioflavin-S staining showing the lack of amyloid properties of the deposits found in the submeningeal level of the cerebral cortex (B). PMCA of blood collected at 140 day post infection (d.p.i.) from a symptomatic animal injected with PMCA-#4. RML dilutions (10^−10^ and 10^−11^) were used in PMCA to estimate the concentration of circulating infectious PrP (C). Biochemical analysis of the brains harvested from the first two animals injected with PMCA-#4 (sacrificed at terminal stage of the disease) were performed and compared to that of RML injected mice (D). Scale bar in A is 10 μm; scale bar in B is 5 μm. Western blots were performed using 6D11 monoclonal antibody to PrP (0.2 μg/mL, Covance). Blots were developed with the enhanced chemiluminescent system (ECL, Amersham Biosciences) and visualized using a G:BOX Chemi Syngene system.

We performed two different transmission studies. In the first one, two groups of CD1 mice (n = 10) were intracerebrally injected with 10% brain homogenate obtained either from the animal injected with preparations #4 or PBS. In the second transmission study, we wanted to assess whether PMCA-#4 possessed infectious properties. In this case two groups of 10 animals were intracerebrally injected with either PMCA-#4 or PMCA-PBS. At the time of writing, no animals of the first study injected with preparation #4 show clinical signs of disease (430 days post inoculation). All animals of the second study injected with PMCA-#4 showed an incubation and survival time of 130 ± 4 and 160 ± 3.85 days (mean ± Standard Error of the Mean, SEM) (Fig 9). The brains of two terminally sick animals from this group were collected and used for biochemical and immunohistochemical analysis. As shown in Fig 10A the animals showed widespread PrP^Res^ deposition, prevalently affecting the hippocampus, thalamus and striatum with plaque-like deposits mainly observed in the submeningeal region of the cerebral cortex and occasionally found in the striatum (Fig 10A). Thioflavin-S (ThS) staining revealed that these deposits did not possess the amyloid tintorial properties (Fig 10B). RML injected mice showed typical synaptic and diffuse PrP^Res^ deposition without the presence of plaque-like deposits that were observed in PMCA-#4 injected animals. Severe spongiform changes were detected in the hippocampus and dorsal medulla of PMCA-#4 infected animals (Figs 10B and 9C) while few vacuoles were found in the rest of the brain, including cerebral cortex and cerebellum (Figs 10A and 9C). On the contrary, RML injected mice showed the highest spongiform changes in the thalamus, septum and cerebral cortex (Fig 9D). Biochemical analysis showed the presence of a PK resistant PrP with a glycoform profile similar to that of the inoculum (PMCA-#4) and characterized by a prevalence of the diglycosylated band (Fig 10C). RML injected mice were characterized by the presence of a typical PrP^Res^ characterized by a predominant monoglycosylated band (Fig 10D). The blood of a PMCA-#4 symptomatic animal, collected at 140 days post infection, was analyzed by means of PMCA and showed the presence of a PK-resistant PrP in the second round of amplification which was characterized by a prevalence of the diglycosylated band (Fig 10C). Using the RML as positive control for PMCA, we could estimate that the concentration of infectious prion circulating in blood is similar to that of a 10^−10^/10^−11^ dilution of brain homogenate.

## Discussion

The production of synthetic prions was introduced in 2004, via a simple *in vitro* induction of misfolding and aggregation of bacterially expressed recPrP [15]. PrP amyloids possessing different conformation stability were generated by altering the conditions for their formation, including urea concentration, pH and temperature. After passaging in mice, a large ensemble of synthetic prions showed a direct relationship between stability and incubation time of novel prion strains [6]. The recent, impressive progress in this technique has spurred the renewed investigation of prion structural biology [41–45].

The aggregation pathway plays an important role in prion disease as it is commonly accepted that both species barrier and strain phenomenon are due to different conversion pathways [46]. However, the molecular basis of prion conversion remains unclear, especially the varied structural landscape of PrP^Sc^, which forms the basis of the strain phenomenon [47]. Therefore, the differences in conformational amyloid states of putative infectious materials—which can be generated *in vitro* under defined biophysical and biochemical conditions using recPrP—are the key elements to determine the biological activities of functional, pathological amyloid fibrils. In our studies, we created amyloid fibers under an array of different chaotropic conditions at two pH values: either mimicking the extracellular environment (neutral pH) or the endocytic compartment (acidic pH at 5). Although the high concentrations of chaotropic agent are not close to physiological conditions, in these cases the kinetics of full-length PrP showed the tendency of the protein to adopt different folding states, which may encipher alternative pathogenic states (so-called prion states). In the presence of Gdn-HCl, our data showed a direct correlation between protein concentration and the final morphologies in fibrillizations (S3B Fig).

Although most fibrillizations were obtained in 72 hours and the stable kinetics of some amyloid preparations was long, the final samples of some fibrillizations visualized under AFM showed oligomeric morphologies (Figs 2 and S3A). Indeed, several oligomerization pathways of PrP may coexist, underlying that some oligomeric types may eventually assemble into fibrils, whereas others may just lead to a dead-end pathway [48, 49].

Earlier works reported that, in infected brains, PrP^Sc^ accumulates at the plasma membrane and occasionally in late endosome/lysosome-like structures [50]. More recent studies show that prion conversion occurs in the endosomal recycling compartment where it transits after being internalized from the cell surface [51]. In addition, in both REDOX and non-REDOX processes, under similar concentration of denaturant and ionic strength, at either neutral pH or acidic pH, a shorter lag phase (S2 Fig) indicated that recMoPrP(23–231) is prone to convert to amyloid forms under neutral pH. On the other hand, amyloid fibrils converted in neutral pH showed low stability when treated with protease and denaturant. In fact, studies have showed that, in mice, less stable amyloids produced less stable prion strains, exhibiting short incubation time [6]. Generally, they replicate faster because of the lower stability. These data suggested that the very first step for PrP conversion and spreading may occur at extracellular sites. Tanaka *et al*. showed that infection of yeast with different amyloid conformations composed of a recombinant Sup35 fragment leads to different [*PSI* ^+^] strains. This evidence indicates that this prion protein adopts an infectious conformation before entering the cells [10].

In general, prion diseases are known to be triggered by PrP conformational conversion and subsequent aggregation [52]. These aggregates may be determined by non-covalent hydrophobic interactions and/or intermolecular disulfide bond formation. The oxidized and reduced states are two basic states of PrP, which are responsible for the formation of disulfide bonds. Starting from these two states, our amyloid preparation processes revealed different formation mechanisms. In the non-REDOX process, the oxidized recMoPrP at high concentration of denaturant was diluted directly into the fibrillization buffer in order to reach the final concentrations. During this procedure, protein particles seem to take a random pathway and aggregate. In this case the size of the clustered aggregation sometimes can be larger than those held together by regular, weaker forces, and may thus cause precipitation [53]. The underlying kinetic mechanism is likely to be a diffusion-limited aggregation process [54]. Indeed, at 3M Gdn-HCl at non-REDOX condition after 72 hours of 15-minutes interval shaking (S9 Fig), we achieved a classical form of diffusion-limited aggregation, whereas this was not obtained for condition #28 (REDOX).

Despite sharing the same conversion mechanism based on domain swapping and rearrangement of disulfide bridge, our REDOX amyloid preparation process differs from one previously described [24] in which His-tag hamster PrP(90–231) was converted into oligomeric forms and showed seed conversion properties in cell-free conversion systems. Indeed, in some cases our REDOX amyloid preparations exhibited a PK-resistant PrP band with a molecular mass of ~19 kDa, as well as characteristics similar to amyloid preparations obtained using a non-REDOX process. These results depart from data reported by Lee S. et al [24].

Considering all these data, it seems that the amyloid fibril prepared under non-REDOX condition (#4) is more prone to coerce the misfolding of PrP^C^ when assessed either *in vitro* or *in vivo*. Notably, the glycoform ratio of this amyloid was tightly maintained in PMCA that was seeded with different source of infectious preparation: (i) raw fibrils preparation, (ii) N2a or GT1 infected cell lysate, (iii) brain homogenate of infected animals. In fact, a vCJD-like glycoform ratio, with a predominance of the diglycosylated band of PrP was always observed. This might be a potential pitfall of the amplification procedure when using a synthetic material, which does not possess an intrinsic original glycosylation pattern. In fact, it has been widely reported that PMCA maintains the biological and infectious properties of the prion strain used as inoculum. In this case, the glycosylation profile of PMCA-#4 might reflect the normal glycosylation pattern of PrP^C^ that was used as substrate for amplification (S11 Fig). When injected in animals, amyloid #4 and #28 did not produce any evident prion pathology. Thus, we have analyzed the CNS of these animals by means of PMCA. After two rounds of PMCA we could detect the presence of a PK-resistant PrP in the brain of a mouse injected with the preparation #4, while we have never detected any PK-resistant protein in the brain of the animals injected with preparation #28 (even after 6 round of PMCA). In this case, we cannot rule out the presence of infectious material in the CNS of mice inoculated with preparation #28 since it could be a new PK-sensitive prion isolate that we cannot detect with our analytic techniques based on PK treatment of the samples.

Here we show that seeds derived from our recMoPrP(23–231) amyloid fibrils, which were generated by an array of recMoPrP(23–231) amyloid preparations, exhibited different structural properties due to different mechanisms of prion aggregation. When directly added to neuronal cell lines, endogenous PrP^C^ was induced to change its conformation to PK-resistant PrP forms, which are well-known diagnostic markers for prions. We used immunofluorescence with confocal microscopy to show location and level of PrP within the neuronal amyloid fibril-infected cells at sixth passage. This observation suggested that small amounts of amyloid fibrils, which were added only in the beginning passage (P1), could seed endogenous misfolded PrP and lead it to accumulate at membrane and cytosolic compartments. Therefore, our amyloid-infected cell culture assay using recMoPrP(23–231) to produce synthetic prions may facilitate investigation aimed at unraveling mechanistic steps in prion formation. This method is indeed a useful and time-efficient tool for screening putative infectious materials.

Infection of wild type animals with amyloid #4 (either first and second passage) did not produce any clinical sign of prion disease. This phenomenon could have several explanations. For example, Makarava et al. [55] reported that there is a period of time where the fibrillar rPrP structure is converted to an intermediate and atypical form of PrP^Res^ (deformed templating) before producing the classical PrP^Sc^ which is able to trigger evident pathological changes in mice. Since the atypical PrP^Res^ is characterized by a short PK-resistant core, a preference for a monoglycosylated PrP and inactivity in PMCA, we have digested the brain of mice injected with amyloid #4 and #28 (first passage) with different concentrations of PK and used anti PrP antibodies which recognize more C-terminal fragments of PrP (SAF-84, residues 160–170; SAF-61 and W226 antibodies which recognize residues 144–152). Even with these modifications we could never detect any typical or atypical PK resistant PrP. Another explanation for the lack of pathological changes in mice injected with amyloid #4 and #28 could be attributable to the small amount of infectious material used to challenge the animals. In this case, the amount of amyloids could not have been sufficient to misfold enough PrP^C^ and induce neuropathological or clinical alterations. This point is supported by our PMCA experiments where we observed that the brain of mice injected with amyloid #4 (first passage) culled at the end of their life-span showed a PrP^Res^ signal after 3 rounds of amplification. When injected in wild type mice, this amplified product (PMCA-#4) was found to be highly aggressive with an attack rate of 100%. In particular, incubation and survival time were 130 ± 4.4 and 160 ± 3.85 days (mean ± standard error of the mean), respectively. The analysis of brains collected from two animals confirmed the presence of PK resistant PrP with distinctive biochemical and neuropathological features. Specifically, the PrP^Res^ showed an uncommon migration profile (compared to known prion strains) with a prevalence of the di-glycosylated band. Neuropathological analysis revealed a synaptic pattern of PrP deposition with few focal deposits (confined to the submeningeal level of the cortex) negative for ThS staining. Finally, this PK-resistant PrP was detected in blood (plasma fraction) collected at 140 days post injection thus demonstrating the aggressiveness of the new prion isolate.

The data we gathered lead us to four important conclusions. Firstly, putative infectious materials can be generated *in vitro*, under controlled and well-defined biophysical and biochemical conditions using solely recPrP and some simple chemicals, without employing prion-infected brain homogenate or purified PrP^Sc^. Secondly, the PK-resistant ability of amyloid fibril-infected cells was maintained during cellular passages. Thirdly, different structural properties of putative infectious materials may account for different prion characteristics. Fourthly, we observed that the amyloid preparation produced *in vitro* (#4) maintained the same biochemical profile when amplified directly from (i) the synthetic preparation or (ii) the infected cell lysates and finally (iii) from the brain of infected mouse. Overall, the production of different batches of the amyloid (#4) maintained the same biochemical and infectious properties when inoculated in cells and challenged by means of PMCA. Nevertheless, after PMCA amplification, this newly generated synthetic prion was found to induce a severe prion pathology when injected in mice thus confirming that the synthetic material is stable and infectious.

## Materials and Methods

### Ethics statement

Mice were housed in groups of 2–5 animals in individually ventilated cages, daily fed and water provided ad libitum. Lighting was on an automatic 12 h basis. Regular veterinary care was daily performed for assessment of animal health. Animal facility is licensed and inspected by the Italian Ministry of Health. Current animal husbandry and housing practices comply with the Council of Europe Convention ETS123 (European Convention for the Protection of Vertebrate Animals used for Experimental and Other Scientific Purposes; Strasbourg, 18.03.1986); Italian Legislative Decree 26/2014, Gazzetta Ufficiale della Repubblica Italiana, 26 July 2014; and with the 86/609/EEC (Council Directive of 24 November 1986 on the approximation of laws, regulations and administrative provisions of the Member States regarding the protection of animals used for experimental and other scientific purposes).

The study, including its Ethics aspects, was approved by the Italian Ministry of Health (Permit Number: NP-02–14). All surgery was performed under tribromoethanol anesthesia, and all efforts were made to minimize suffering.

### Expression and purification of recPrP

RecMoPrP(23–231) was expressed in *E*. *coli* Rosetta2(DE3) with pET11a(MoPrP23-231)-without His-tag. The transformed bacteria were grown in Luria Bertani (LB) media using a Biostat B plus fermenter 2L vessel. Expression was induced with Isopropyl β-D-1-thiogalactopyranoside (IPTG) at a final concentration of 1mM. The cultures were harvested after 24 hours of induction, centrifuged (1500 x g, 30min, 4°C) and resuspended in buffer A (25mMTris-HCl pH 8.0, 5mM Ethylenediaminetetraacetic acid (EDTA), 1mM phenylmethylsulfonyl fluoride (PMFS)). After centrifugation, pellets were resuspended in buffer B (25mMTris-HCl, 5mM EDTA, 1mM PMSF, 0.5% Triton X-100). To disrupt bacterial cells, the solution was passed three times through the microfluidizer at 15000–18000 psi. The solution was centrifuged for 30min at 3400 x g and inclusion bodies were washed twice with buffer C (25mMTris-HCl, 5mM EDTA, 0.8% Triton X-100) and buffer D (25mMTris-HCl pH 8.0) and twice with double-distilled H_2_O. Pellets containing MoPrP(23–231) protein were solubilized in 8M Gdn-HCl, shaken overnight at 37°C and centrifuged (3400 x g, 30min). The solution was then brought to buffer containing 6M Gdn-HCl, 20mMTris-HCl pH8.0, 500mM NaCl and loaded onto HisTrap FF crude column (GE Healthcare). The column was washed with buffer A (20mM Tris-HCl pH 8.0, 10mM Imidazole, 2M Gdn-HCl) and protein was eluted with linear imidazole gradient (20–500 mM imidazole in buffer A). Fractions containing MoPrP(23–231) protein were then loaded onto reverse phase column (Jupiter C4, 250x21.2mm, 300 A, Phenomenex). The column was washed with buffer A (0.1%TFA) and protein was eluted with a linear gradient from 0 to 95% acetonitrile in 0.1% Trifluoroacetic Acid (TFA) (buffer B). Samples containing PrP protein were then lyophilized.

### Amyloid preparations

RecMoPrP(23–231) was expressed and purified as described. All stock solutions for fibrillization were sterile, filtered through a 0.22μm filter prior to each assay in order to avoid the presence of contaminants. Lyophilized protein was dissolved in 6M Gdn-HCl at 10mg/mL or 8M Urea at 10mg/mL, aliquoted, and frozen at -80°C. To form fibrils in the non-REDOX process, a solution of Gdn-HCl (concentrations are indicated in Table 1), 50mM buffer acetate pH 5.5 or PBS pH 7.5, NaCl (concentrations are indicated in S1 Table) and 10 μm Thioflavine T (ThT) was mixed before adding recPrP, which has a final concentration of 100 μg/mL or 200 μg/mL (indicated in S1 and S2 Tables). To form fibrils in the REDOX process as described (Fig 1), after dissolving in 6M Gdn-HCl, the lyophilized protein was reduced by adding 100mM Dithiothreitol (DTT) at 37°C for 1 hour. In case of conditions containing NaCl (as indicated in S2 Table) this was added to the protein stock solution saturated level. The next steps were the same as those of the non-REDOX process described above. For fibrillization, a 3-mm glass bead (Sigma) was added to each well of a 96-well black plate with clear bottom (BD Falcon). The final volume protein solutions were added to 200 μL/well. The plate was covered with sealing tape (Fisher Scientific) and shaken continuously at 37°C using M5 fluorescence plate reader with automix capability (Spectramax M5 Molecular Devices. ThT fluorescence was measured with the same plate reader at 444/485 nm excitation/emission spectra every 5 min after 72 hours or 52 hours continuous shaking by bottom fluorescence reading. Each sample was measured in six independent replicate wells. Fibrils were collected by ultracentrifugation at 100,000 x g for 30 min to remove other soluble components before further characterizations.

### Atomic force microscopy (AFM)

This method was employed in accordance with that described above [31, 56]. Specimen were imaged with a Nanowirzard-II BioAFM (JPK Instruments AG, Berlin, Germany, www.jpk.com) operating in dynamic mode and using non-contact Si cantilevers (NSG11, NT-MDT–Moscow, Russia, www.ntmdt.com or ARROW-NCR, Nano World-Neuchȃtel, Switzerland, www.nanoworld.com) with tip radii of <7–10 nm, spring constants of 20–40 N/m, and resonance frequencies of 285–325 kHz. After fibrillization, 5–10μL samples were spread onto a freshly cleaved mica sheet and left to adhere for 10-20min. Samples were then washed with distilled H_2_O and dried naturally. The images were acquired at line scan rates of 0.5–1 Hz at room temperature (RT). The AFM free oscillation amplitudes ranged from 25nm to 40nm, with characteristic set points ranging from 75% to 90%. AFM data were analyzed with Gwyddion (www.gwyddion.net).

### Testing for disulfide bond interchain of fibrils from REDOX process

After fibrillizations, 1μg of protein samples was precipitated by ultracentrifugation at 100,000 x g for 30min, dissolved in non-reducing sample buffer (125 mM TrisHCl, pH 6.8, 4% SDS, 0.2% bromophenol blue, 20% glycerol) and separated in 10% non-reducing SDS-PAGE gels. For disulfide bond interchain test, 1μg of protein after precipitation by ultracentrifugation was treated with (DTT) at the last concentration of 150mM or incubated in 1mL 6M Gnd-HCl for 3 days, then treated with 150mM DTT. These samples were centrifuged at 100,000 x g for 1 hour, resuspended in reducing loading buffer, boiled for 10min at 100°C and separated in 10% polyacrylamide gels. Western blotting was carried out and gels were subsequently transferred overnight onto Immobilon P PVDF membranes (Millipore). Membranes were blocked by 5% non-fat milk, incubated first with 1 μg/mL anti-PrP Fab D18 and then with goat anti-human IgG F(ab)2 fragment conjugated with horseradish peroxidase (HRP). Blots were developed with the enhanced chemiluminescent system (ECL, Amersham Biosciences) and visualized on Hyperfilm (Amersham Biosciences).

### Preparations of prion amyloid fibrils for cellular assays

Collection of amyloid fibrils from 96-well plates into 1.5 mL tubes was performed under sterile conditions. Samples were centrifuged at 100,000 x g for 30 min at 4°C in an ultracentrifuge (Beckman Coulter). The pellets were resuspended in sterile PBS and then sonicated for 5 min prior to being added to cultured cell media.

### Cell cultures

Mouse neuroblastoma N2a cells were cultured in minimal essential medium with Earle’s salt (EMEM) supplemented with 10% FBS, 1% non-essential amino acids, 1% L-glutamax, 100 units/mL penicillin and 100 μg/mL streptomycin. Mouse hypothalamic GT1 cells and mouse hippocampal PrP-deficient HpL3-4 were cultured in Dulbecco’s modified Eagle’s medium (DMEM) supplemented with 10% fetal bovine serum (FBS), 100 units/mL penicillin and 100 μg/mL streptomycin. Cell cultures were cultivated in 10-cm plates and incubated at 37°C in humidified 5% CO_2_. Cells were split at ratio 1:10 for further cultivation when the confluence reached 95%.

### Infecting N2a, GT1 and HpL 3–4 cell lines with amyloid fibrils

At the end of the fibrillization, after collection as describe above, 200μL of fibril solutions were resuspended in sterile PBS and sonicated in a water-cooled cup-horn sonicator for 5min for preparation of fibrillar infections. Cells were seeded in tissue culture plates 1 day prior to infection and around 5% confluent on the day of the infection. After removing the media, cells were washed and changed once with fresh media. After adding fibrils into the media of cells, the incubation was done over 6-7days. Once confluency was reached, cells in the plate were washed with PBS, trypsinized and transferred to another tissue culture plate. Cells were subjected to 1:10 split, which was counted as passage 1. Subsequently, cells were subjected to routine cell culture procedures for further passages. Cell lysates were collected at every passage. After the cell plates reached the confluency, they were washed with PBS and lysed in 500 μL cell lysis buffer (10mMTrisHCl pH 8.0, 150mMNaCl, 5.0% NP40, 5.0% deoxycholic acid sodium salt (DOC)). Total protein content of cell lysates was measured using bicinchoninic acid (BCA) protein quantification kit (Pierce) and stored at -20°C until analysis.

### PK-resistant PrP detection in amyloid fibril-infected cell lysates by western blotting

Fibril-infected cell lysates were collected at each passage. The accumulation of PK-resistant PrP was detected by proteinase K (PK) digestion followed by immunoblotting of lysated cells. Five hundred μL of lysis buffer (10 mM Tris-HCl pH 8.0, 150 mM NaCl, 0.5%, NP40, 0.5% DOC) was added to each 10-cm cell plate and cell lysates were collected after centrifugation at 400 x g for 5 min in a bench microfuge (Eppendorf). The total protein content of samples was measured by means of bicinchoninic acid assay (BCA) (Pierce). Cell lysated samples were adjusted to 1mg/mL total protein; 2μg/mL PK (Invitrogen) was added to reach the final volume of 0.5 mL. Following 1 hour incubation at 37°C, digestion was stopped by adding PMSF at 2mM final concentration. Digestion products were precipitated by centrifugation at 100,000 x g for 1 hour at 4°C in an ultracentrifuge (Beckman Coulter), and resuspended in 2X SDS-PAGE loading buffer (125 mM TrisHCl, pH 6.8, 10% 2-mercapethanol, 4% SDS, 0.2% bromophenol blue, 20% glycerol). For non-PK digested samples, 50 μg of cell lysates were used and 2X SDS-PAGE loading buffer was added in a 1:1 ratio. The samples were boiled for 10 min at 100°C, loaded onto a 12% Tris-Glycine SDS-PAGE gel, and transferred overnight onto Immobilon P PVDF membranes (Millipore). Membranes were blocked by 5% non-fat milk, incubated first with 1 μg/mL anti-PrP Fab D18, Clone P and then with goat anti-human IgG F(ab)2 fragment HRP-conjugated. Blots were developed with the enhanced chemiluminescent system (ECL, Amersham Biosciences) and visualized on Hyperfilm (Amersham Biosciences).

### PK-resistant PrP detection in different amyloid preparations by western blotting

After fibrillization, 100 μL samples were centrifuged at 100,000 x g for 30 min at 4°C (Beckman Coulter ultracentrifuge). Pellets were resuspended in the same volume of PBS. The samples were then digested by adding either 2 μg/mL or 20 μg/mL of PK in PBS for 1 hour at 37°C. The reaction was stopped with 2 mM PMSF and the PK-digested samples were centrifuged at 100,000 x g for 1 hour at 4°C (Beckman Coulter ultracentrifuge). Pellets were resuspended in 1X SDS-PAGE loading buffer. The non-PK digested samples of fibril solutions were added into 2X SDS-PAGE loading buffer in a 1:1 ratio. The samples were boiled for 10 min at 100°C, loaded onto a 12% Tris-Glycine SDS- PAGE gel, and transferred overnight onto Immobilon P PVDF membranes (Millipore). Membranes were blocked by 5% non-fat milk, incubated first with 1 μg/mL anti-PrP Fab D18 and then with goat anti-human IgG F(ab)2 fragment HRP-conjugated. Blots were developed with the enhanced chemiluminescent system (ECL, Amersham Biosciences) and visualized on Hyperfilm (Amersham Biosciences).

### Fluorescence immunostaining of prion amyloid fibril-infected cells

One million cells/mL of each cell line were cultured for 1 day in each well of a 24-well plate containing a 1.2 cm-coverslip and appropriate culture medium. Medium was removed and cells were washed with PBS. Cells were fixed for 30min in 4% PFA (paraformaldehyde in PBS) at RT then washed twice for 15 min with PBS. Fixed cells were blocked in blocking buffer (5% normal rabbit serum (NRS) in PBS + 0.3% Triton), exposed to anti-PrP monoclonal antibody Fab D18 or clone P (10 μg/mL final concentration) for 2 hours. Primary antibodies were made up in 1% blocking solution and PBS. Cells were washed once with PBS for 15min. The secondary antibody used is goat anti-human Alexa 488 at 1:500 dilution in 1% blocking buffer and PBS. Secondary antibodies were incubated for 1 hour at RT. Finally, cells were washed twice for 15min with PBS. The coverslips were taken out and dried naturally. The nuclei (blue) were stained with DAPI, coverslips were mounted in mounting media and subsequently placed on glass slides and stored at 4°C for confocal fluorescence microscopy.

### Measurement of cell viability

Cell viability was examined based on mitochondrial activity, measured by MTT assay. GT1 and N2a cells were maintained in DMEM and EMEM, respectively, and supplemented with FBS and antibiotics. After 1 day of incubation, media were aspirated from a confluent 10-cm plate, and cells were detached by adding 1 mL of 1X trypsin-EDTA solution. Media were added, and cell density determined using a haemacytometer. Cell density was adjusted to 2.5 × 10^5^ cells/mL with media. A 96-well, tissue culture-treated, clear bottom, black plate (Costar) wetted with 96 μL of media was incubated at 37°C, prior to use. One hundred microliters of cell suspension were added to each well and cells were allowed to settle for 2 hours, before adding prion amyloid fibrils. Four microliters of prion fibril solutions from different amyloid preparations in PBS were added to each well (final concentration 2μg/mL of PrP), and plates were incubated at 37°C in 5% CO_2_. After incubating for 5 days, media were aspirated and cells were washed twice with 200 μL of PBS. After treatment with different amyloid samples, cells were further incubated for 3 hours in fresh media with a final concentration of 0.5 mg/mL MTT. DMSO was added to release the insoluble purple substrates converted by the active mitochondrial dehydrogenases in the surviving cells. After shaking 30min for solubilization, the adsorbance was measured using a SpectraMax M5 plate reader [57].

### Intracerebral inoculation of amyloid preparations #4 and #28 (first passage)

Twenty μl of each amyloid preparation (100 μg of total fibrils) was stereotactically injected in the striatum of otubred CD1 mice (n = 8). Six week-old CD1 mice (35–40 g) were anesthetized with tribromoethanol (100 μL/10 g) i.p.-administered and all surgical procedures were performed under sterile conditions. All the animals were sacrificed at 720 days post-inoculation.

### PMCA procedures

PMCA was performed as previously described [51]. Briefly, as a substrate, we used brain specimens obtained from outbred CD1 animals. Brains were harvested after perfusion, and 10% homogenate was prepared in conversion buffer (PBS containing 150 mM sodium chloride and 1% Triton X-100) with the addition of protease inhibitors. Ten μl of raw fibril preprations, cell lysates from infected cells (P6) or brain homogenates from infected mice were added to the substrate and transferred in 0.2 mL tubes, positioned on an adaptor placed on the plate holder of a microsonicator (Misonix, Model S3000) and subjected to 96 cycles of PMCA. Each cycle (also referred as PMCA round) consisted of 29 min and 40 sec of incubation at 37/40°C followed by a 20 sec pulse of sonication set at potency of 260–270 Watt. After one round of PMCA, an aliquot of the amplified material was diluted 10-folds into fresh substrate and a further PMCA run performed following the same procedure. To increase PMCA efficiency, teflon beads (n = 3) were added to the samples before each round of amplification. To avoid samples cross-contamination between each round, thorough decontamination of instruments and equipment was performed using 2N sodium hydroxide (NaOH) or 4M guanidine hydrochloride (Gdn-HCl).

### Neuropathological analysis

Brains and other organs were fixed in Alcolin (Diapath), dehydrated and embedded in paraplast. Seven-micrometer thick serial sections were stained with hematoxylin-eosin (H&E) and thioflavine S, or immunostained with monoclonal antibodies to PrP (6H4), and polyclonal antibodies to astrocyte activation (GFAP) and microglial activation (Iba-1). Before PrP immunostaining the sections were sequentially treated with proteinase K (10 μg/mL, 5 min) and guanidine isothiocyanate (3M, 20 min), and non-specific binding was prevented using ARK kit (Dako). Immunoreactions were visualized using 3–3'-diaminobenzidine (DAB, Dako) as chromogen. Lesion profile was performed according to Fraser H. et al 1968 [58].

### Biochemical analysis of brain homogenates

Ten percent (wt/vol) brain homogenates from frozen tissues were prepared in lysis buffer. The cleared lysate (20 μL) was digested with 50 μg/mL of proteinase K (1 h, 37°C). Proteins were boiled (100°C, 10 minutes) then fractionated using 12% SDS-PAGE under reducing condition and transferred into nitrocellulose membranes. Immunoblotting with 6D11 (Covance) diluted in TBST (0.2 μg/mL), 0.05%-Tween20 was performed. Blots were developed using the ECL Prime detection system (Amersham) and visualized using a G:BOX Chemi Syngene system.

### PNGase experiment

Fifty μl of brain homogenates were digested with PK (100 μg/mL) for 1 hour at 37°C with shaking. PNGase digestion was performed following the instructions provided with the kit (PNGase F, P0704S, 15.000 units, New England Biolabs). Briefly, 4 μl of 10X glycoprotein denaturing buffer (provided with the kit) were added to the homogenate and the samples were boiled for 10 minutes at 100°C. Subsequently, 4 μl of G7 buffer (provided with kit), 4 μl of NP-40 (provided with kit), 4 μl of PNGase F and 4 μl of PBS were added to reach the final volume of 40 μl. Each sample was incubated 2 hours at 37°C with shaking. The reaction was stopped by adding NuPAGE LDS sample buffer and Western blotting was performed.

### Second passage transmission

Twenty μl of brain homogenate from the animals injected with preparation #4 was stereotaxically inoculated in the striatum of outbred CD1 mice (n = 10). Six week-old CD1 mice (35–40 g) were anesthetized with tribromoethanol (100 μl/10 g) i.p.-administered and all surgical procedures were performed under sterile conditions

## Supporting Information

S1 Table Conditions used for the formation of diverse amyloid preparations in non-REDOX process. To generate recPrP amyloids with different conformations in non-REDOX process, the conditions for their formation were systematically altered, including denaturant concentrations, pH and buffer composition.
(DOCX)

S2 Table Conditions used for the formation of diverse amyloid preparations in REDOX process. To generate recPrP amyloids with different conformations in REDOX process, the conditions for their formation were systematically altered, including denaturant concentrations, pH and buffer composition.
(DOCX)

S3 Table Different height of clusters of recMoPrP (23–231) aggregates. Different amyloid preparations of recMoPrP(23–231) showed aggregate clusters of different heights.
(DOCX)

S1 Fig Diagram of recMoPrP(23–231) conversion to amyloid. Schematic diagram for the conversion of the monomeric recMoPrP(23–231) to an amyloid form by REDOX process.
(TIF)

S2 Fig Lag phase distribution using different conditions (REDOX *vs* non-REDOX). Lag phase distribution of amyloid preparations following non-REDOX and REDOX processes compared to pH 5.0 and pH 7.5. (**, P<0.01, n = 12). Bars indicate standard deviation.
(TIF)

S3 Fig AFM characterization of different synthetic amyloid fibrils. The morphology-dependence of the fibrillizations was observed at different denaturant concentrations. AFM imaging at the end of the fibrillization reactions shows different morphologies of amyloid preparations after 72 hours of fibrillization (**A**). Correlation of amyloidal morphologies and Gdn-HCl concentrations in fibrillizations (**B**).
(TIF)

S4 Fig PK digestion assay of different amyloid preparations. Western blotting of PK digestion assay showed partial protease K (PK) resistance of recMoPrP(23–231) amyloid preparations from non-REDOX (**A**) and REDOX (**B**). RecMoPrP(23–231) amyloids (PK- lanes) were digested with PK at ratio 1:10 (w/w) (PK+ lanes) and 1:1 (w/w) (PK++ lanes). Western blots were performed using Fab D18 monoclonal antibody (1μg/mL). Blots were developed with the enhanced chemiluminescent system (ECL, Amersham Biosciences) and visualized on Hyperfilm (Amersham Biosciences)
(TIF)

S5 Fig Seeding assay of N2a and GT1 cell cultures with synthetic amyloid fibrils. Seeding of recMoPrP(23–231) amyloid preparations induced the conversion of endogenous PrP^C^ into mild protease K (PK) resistant forms. Western blotting shows the partial PK-resistance of neuroblastoma N2a and mouse hypothalamic GT1 amyloid fibril-infected cell lysates. Fibril-infected cell lysates (PK- lanes) were digested with PK at ratio 1:500 (w/w) (PK+ lanes). Western blots were performed using Fab D18 monoclonal antibody (1μg/mL) and Clone P (1μg/mL). Blots were developed with the enhanced chemiluminescent system (ECL, Amersham Biosciences) and visualized on Hyperfilm (Amersham Biosciences)
(TIF)

S6 Fig PrP accumulation in N2a cell lines. Accumulation of PrP was observed in neuroblastoma N2a cell lines infected with different amyloid preparations. The depositions and level of PrP (green) after six passages (P6) were detected by Fab D18 anti PrP antibody (10 μg/mL final concentration), using immunofluorescence. The nuclei (blue) were stained with DAPI.
(TIF)

S7 Fig PrP accumulation in GT1 cell lines. Accumulation of PrP was observed in mouse hypothalamic GT1 cell lines infected with different amyloid preparations. The depositions and level of PrP (green) after six passages (P6) were detected by Fab D18 anti PrP antibody (10 μg/mL final concentration), using immunofluorescence. The nuclei (blue) were stained with DAPI.
(TIF)

S8 Fig MTT assay of N2a and GT1 infected cell lines. Amyloid fibrils of recMoPrP(23–231) showed mild toxicity in infected-cell cultures. Cell viability based on mitochondrial activity was measured by MTT assay. Control cells without treatment were counted as 100%. Bars indicate Standard deviation. *t*-test analysis were performed (*, P>0.05, n = 6).
(TIF)

S9 Fig AFM analysis of amyloid #18. AFM imaging analyses were carried out at the end of the fibrillization reaction of amyloid preparation #18 (non-REDOX) after 72 hours with 15 minutes of interval-shaking time. AFM scan topographical image of PrP amyloid #18 deposited on mica surface showed classical forms of diffusion-limited aggregation.
(TIF)

S10 Fig Immunohistochemical and biochemical assays of amyloid-#4 and amyloid-#28 infected mice (first passage). Neuropathological assessment of CD1 mice injected with preparation #4 or #28. Immunohistochemical analysis revealed the lack of PrP^Res^ deposition in the brain associated to mild astroglial activation which is similar to that of the animal injected with PBS. Scale bar is 10 μm (A). Western blot analysis confirmed the lack of PrP^Res^ deposition in the brain of injected animals. Samples were digested with PK (100 μg/mL) and immunoblotted with 6D11 monoclonal antibody to PrP (0.2 μg/mL, Covance) (B). Blots were developed with the enhanced chemiluminescent system (ECL, Amersham Biosciences) and visualized using a G:BOX Chemi Syngene system.
(TIF)

S11 Fig PrP^C^ biochemical profile in wild type animals. Western blot analysis of PrP^C^ biochemical profile in the brain of CD1 mice (dilutions 1:10 and 1:50). Western blots were performed using 6D11 monoclonal antibody to PrP (0.2 μg/mL, Covance). Blots were developed with the enhanced chemiluminescent system (ECL, Amersham Biosciences) and visualized using a G:BOX Chemi Syngene system.
(TIF)

S12 Fig Infectious properties of new batches of amyloid preparations. To assess whether the infectious amyloid #4 was obtained in a random phenomenon, we have used the same biochemical conditions to produce a new batch of this amyloid (#4) and we have assessed its infectious properties either in cells and by means of PMCA. Atomic Force Microscopy (AFM) imaging analysis of the new batch of amyloid #4 was performed at the end of the fibrillization reactions after 72 hours. AFM scan topographical images of prion protein (PrP) deposited on mica surface, large-scale images (**A**); Independent fibrillization kinetic experiments with ThT as fluorescence dye (**B**); Western blots of amyloid #4 infected cell lysates (N2a and GT1) after PK digestion (2μg/mL) incubated with the Clone P monoclonal antibody (1 μg/mL final concentration) (**C**); Immunofluorescence staining of amyloid #4-infected N2a and GT1 cells using anti-PrP monoclonal antibody Fab D18 (10 μg/mL final concentration) (**D**); Seeding ability of new batch of amyloid #4 (raw fibril) was assessed by means of PMCA using brain homogenates of CD1 mice as substrates for amplification. New batches of amyloid #4, #19 and #28 were inoculated in N2a cells, collected at P6 and amplified in PMCA. Amplification of ScN2a infected cell lysate was used as internal control for PMCA reaction. Western blots were performed using 6D11 monoclonal antibody to PrP (0.2 μg/mL, Covance). Blots were developed with the enhanced chemiluminescent system (ECL, Amersham Biosciences) and visualized using a G:BOX Chemi Syngene system (**E**).
(TIF)
