# Supplementary material for: Synthetic prions with novel strain-specified properties
Source: PLoS Pathog. 2015 Dec 31;11(12):e1005354. doi: 10.1371/journal.ppat.1005354 (PMC4699842; doi:10.1371/journal.ppat.1005354)
Supplement: S1 Table — To generate recPrP amyloids with different conformations in non-REDOX process, the conditions for their formation were systematically altered, including denaturant concentrations, pH and buffer composition. (DOCX) [file ppat.1005354.s001.docx]

S1 Table. Conditions used for the formation of diverse amyloid preparations in non-REDOX process

| **Amyloid preparation(#)** | **[Denaturant]** | **Buffer** | **pH** | **[recMoPrP] µg/mL** | **[NaCl] (M)** |
| --- | --- | --- | --- | --- | --- |
| **1** | 4M Urea | 50mM Acetate | 6.0 | 100 | - |
| **2** | 4M Urea | 50mM Acetate | 5.0 | 100 | - |
| **3** | 4M Urea | 50mM Acetate | 3.5 | 100 | - |
| **4** | 4M Gdn-HCl | 50mM Acetate | 5.0 | 100 | - |
| **5** | 3M Gdn-HCl | 50mM Acetate | 5.0 | 100 | - |
| **6** | 2M Gdn-HCl | 50mM Acetate | 5.0 | 100 | - |
| **7** | 1M Gdn-HCl | 50mM Acetate | 5.0 | 100 | - |
| **8** | 0.5M Gdn-HCl | 50mM Acetate | 5.0 | 100 | - |
| **9** | 0.25M Gdn-HCl | 50mM Acetate | 5.0 | 100 | - |
| **11** | 4M Urea | 50mM Acetate | 5.0 | 100 | 0.4 |
| **12** | 4M Urea | 50mM Acetate | 5.0 | 200 | 0.4 |
| **13** | 1M Gdn-HCl | 50mM Acetate | 5.0 | 200 | 0.4 |
| **14** | 2M Gdn-HCl | PBS | 7.5 | 200 | - |
| **17** | 4M Gdn-HCl | PBS | 7.5 | 100 | - |
| **18** | 3M Gdn-HCl | PBS | 7.5 | 100 | - |
| **19** | 2M Gdn-HCl | PBS | 7.5 | 100 | - |
| **20** | 1M Gdn-HCl | PBS | 7.5 | 100 | - |
| **21** | 0.5M Gdn-HCl | PBS | 7.5 | 100 | - |
| **22** | 0.25M Gdn-HCl | PBS | 7.5 | 100 | - |
