# Supplementary material for: Synthetic prions with novel strain-specified properties
Source: PLoS Pathog. 2015 Dec 31;11(12):e1005354. doi: 10.1371/journal.ppat.1005354 (PMC4699842; doi:10.1371/journal.ppat.1005354)
Supplement: S2 Table — To generate recPrP amyloids with different conformations in REDOX process, the conditions for their formation were systematically altered, including denaturant concentrations, pH and buffer composition. (DOCX) [file ppat.1005354.s002.docx]

S2 Table. Conditions used for the formation of diverse amyloid preparations in REDOX process

| **Amyloid preparation(#)** | | **[Denaturant]** | **Buffer** | **pH** | **[recMoPrP] µg/mL** | **[NaCl]** |
| --- | --- | --- | --- | --- | --- | --- |
| **23** | 1M Gdn-HCl | | 50mM Acetate | 5.0 | 100 | + |
| **24** | 1M Gdn-HCl | | 50mM Acetate | 5.0 | 100 | - |
| **25** | 2M Gdn-HCl | | 50mM Acetate | 5.0 | 100 | + |
| **26** | 2M Gdn-HCl | | 50mM Acetate | 5.0 | 100 | - |
| **27** | 3M Gdn-HCl | | 50mM Acetate | 5.0 | 100 | + |
| **28** | 3M Gdn-HCl | | 50mM Acetate | 5.0 | 100 | - |
| **29** | 4M Gdn-HCl | | 50mM Acetate | 5.0 | 100 | + |
| **30** | 4M Gdn-HCl | | 50mM Acetate | 5.0 | 100 | - |
| **31** | 1M Gdn-HCl | | PBS | 7.4 | 100 | + |
| **32** | 1M Gdn-HCl | | PBS | 7.4 | 100 | - |
| **33** | 2M Gdn-HCl | | PBS | 7.4 | 100 | + |
| **34** | 2M Gdn-HCl | | PBS | 7.4 | 100 | - |
| **35** | 3M Gdn-HCl | | PBS | 7.4 | 100 | + |
| **36** | 3M Gdn-HCl | | PBS | 7.4 | 100 | - |
| **37** | 4M Gdn-HCl | | PBS | 7.4 | 100 | + |
| **38** | 4M Gdn-HCl | | PBS | 7.4 | 100 | - |
