# Supplementary material for: Synthetic prions with novel strain-specified properties
Source: PLoS Pathog. 2015 Dec 31;11(12):e1005354. doi: 10.1371/journal.ppat.1005354 (PMC4699842; doi:10.1371/journal.ppat.1005354)
Supplement: S3 Table — Different amyloid preparations of recMoPrP(23–231) showed aggregate clusters of different heights. (DOCX) [file ppat.1005354.s003.docx]

S3 Table. Different height of clusters of recMoPrP (23-231) aggregates

| **Amyloid preparation (#)** | **Height (nm)** | **Top height distribution (nm)** |
| --- | --- | --- |
| **5** | 0.8-1.2 | 1.6 |
| **6** | 0.5-0.8 | 0.5 |
| **11** | 4 | 4 |
| **14** | 3-4.5 | 4.5 |
| **18** | 1.6-1.8 | 1.6 |
| **20** | 5-7 | 5 |
| **25** | 1.8-2 | 1.6-1.8 |
| **26** | 4-18 | 4 |
| **27** | 1.6-5 | 1.6-1.8 |
| **29** | 40-43 | 40 |
| **30** | 30-35 | 60 |
| **31** | - | 0.35 |
| **33** | 1.5-2.5 | 1.5 |
| **34** | 1.6-2.2 | 1.6 |
