# Supplementary figures and images for: Synthetic prions with novel strain-specified properties
Source: PLoS Pathog. 2015 Dec 31;11(12):e1005354. doi: 10.1371/journal.ppat.1005354 (PMC4699842; doi:10.1371/journal.ppat.1005354)

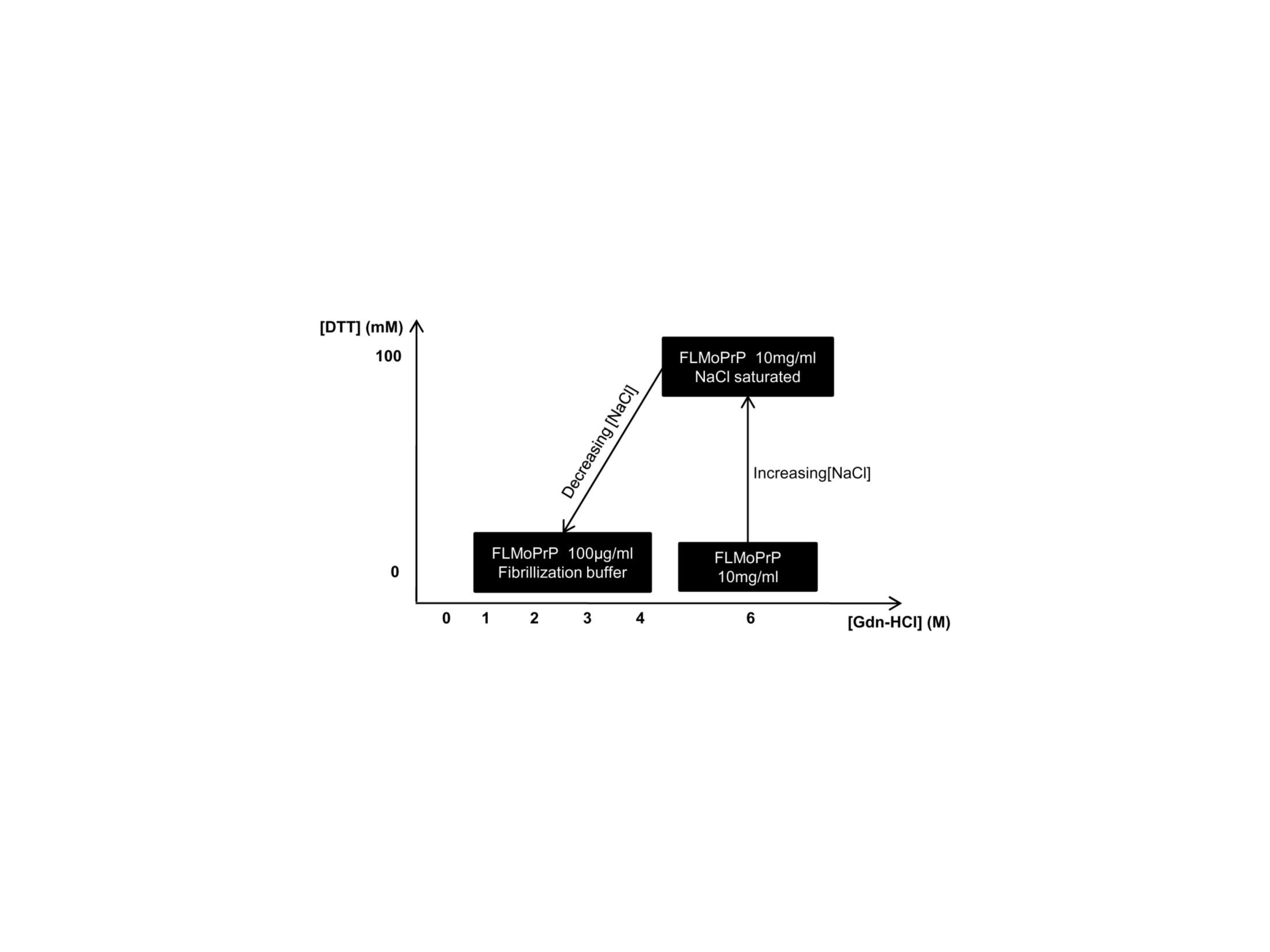

Supplement: S1 Fig — Schematic diagram for the conversion of the monomeric recMoPrP(23–231) to an amyloid form by REDOX process. (TIF) [file ppat.1005354.s004.tif]

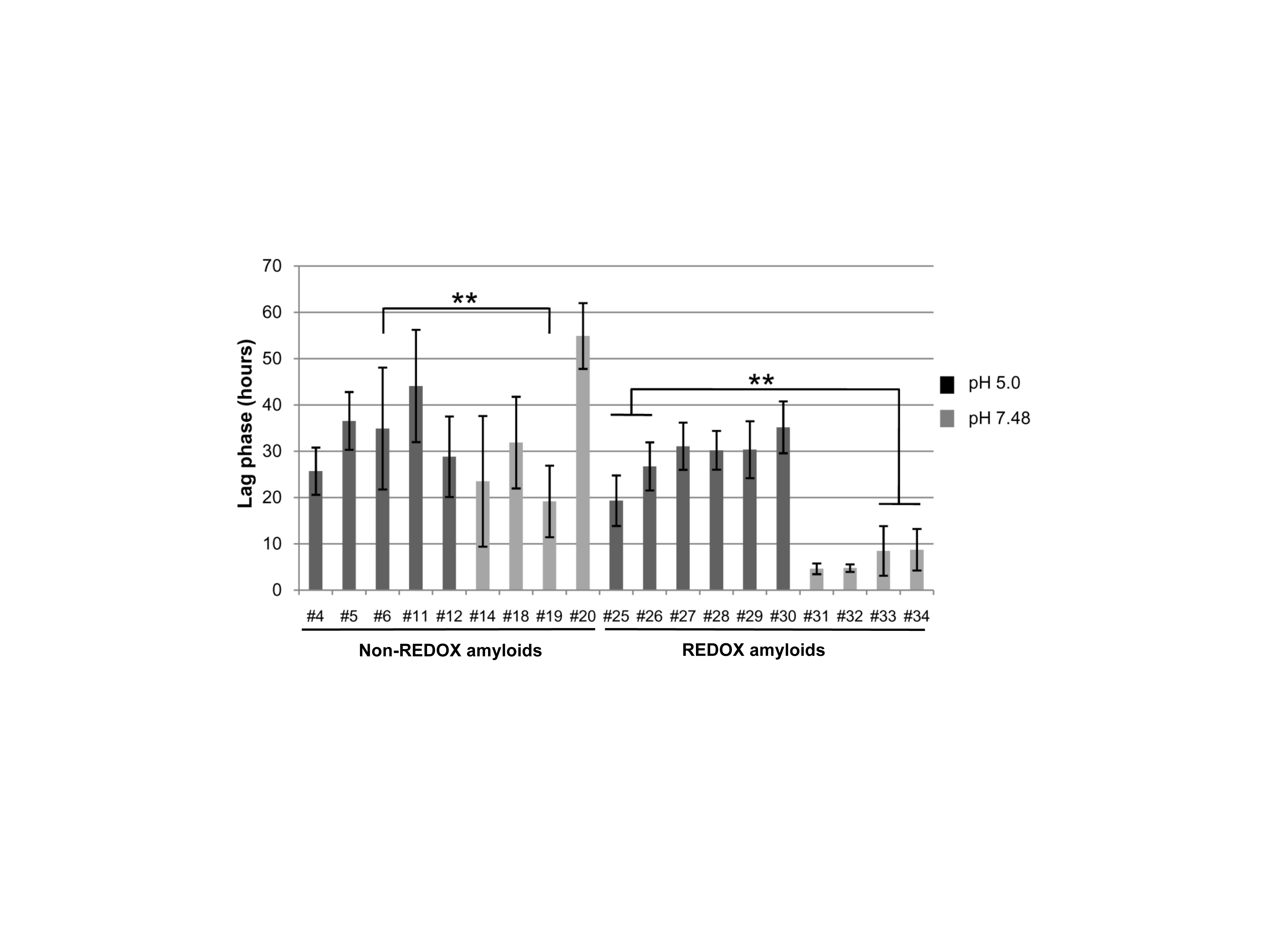

Supplement: S2 Fig — Lag phase distribution of amyloid preparations following non-REDOX and REDOX processes compared to pH 5.0 and pH 7.5. (**, P<0.01, n = 12). Bars indicate standard deviation. (TIF) [file ppat.1005354.s005.tif]

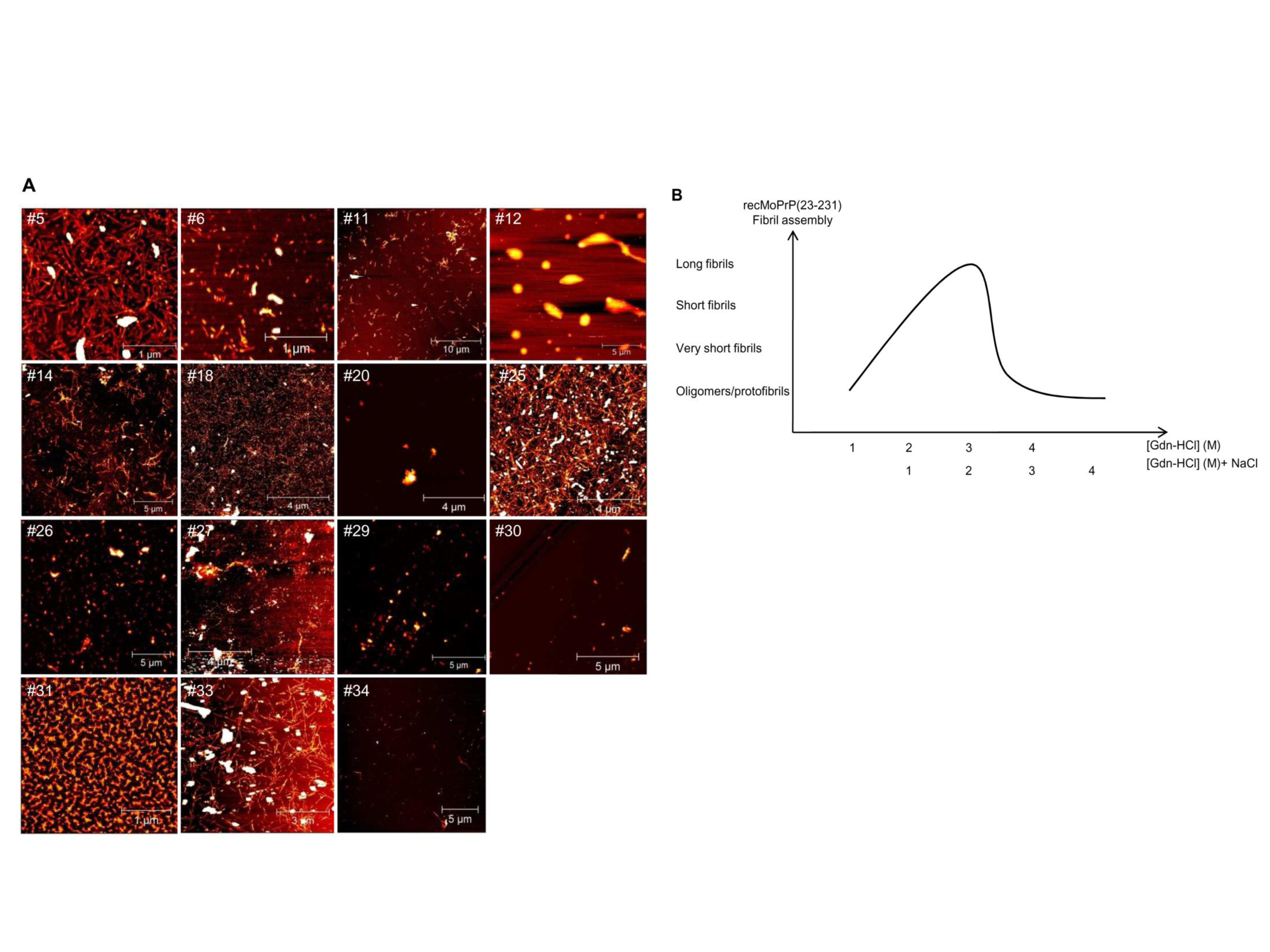

Supplement: S3 Fig — The morphology-dependence of the fibrillizations was observed at different denaturant concentrations. AFM imaging at the end of the fibrillization reactions shows different morphologies of amyloid preparations after 72 hours of fibrillization (A). Correlation of amyloidal morphologies and Gdn-HCl concentrations in fibrillizations (B). (TIF) [file ppat.1005354.s006.tif]

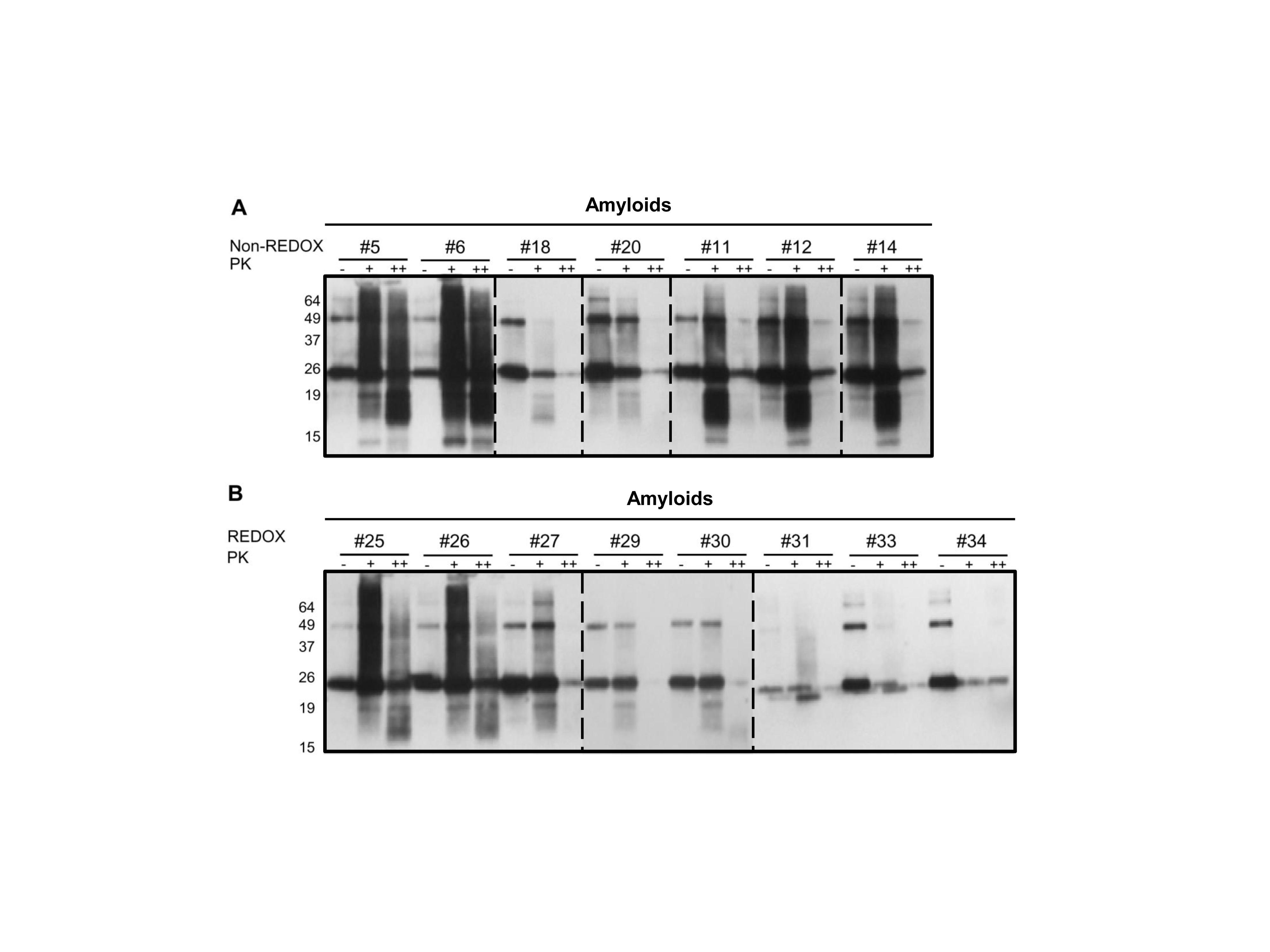

Supplement: S4 Fig — Western blotting of PK digestion assay showed partial protease K (PK) resistance of recMoPrP(23–231) amyloid preparations from non-REDOX (A) and REDOX (B). RecMoPrP(23–231) amyloids (PK- lanes) were digested with PK at ratio 1:10 (w/w) (PK+ lanes) and 1:1 (w/w) (PK++ lanes). Western blots were performed using Fab D18 monoclonal antibody (1μg/mL). Blots were developed with the enhanced chemiluminescent system (ECL, Amersham Biosciences) and visualized on Hyperfilm (Amersham Biosciences) (TIF) [file ppat.1005354.s007.tif]

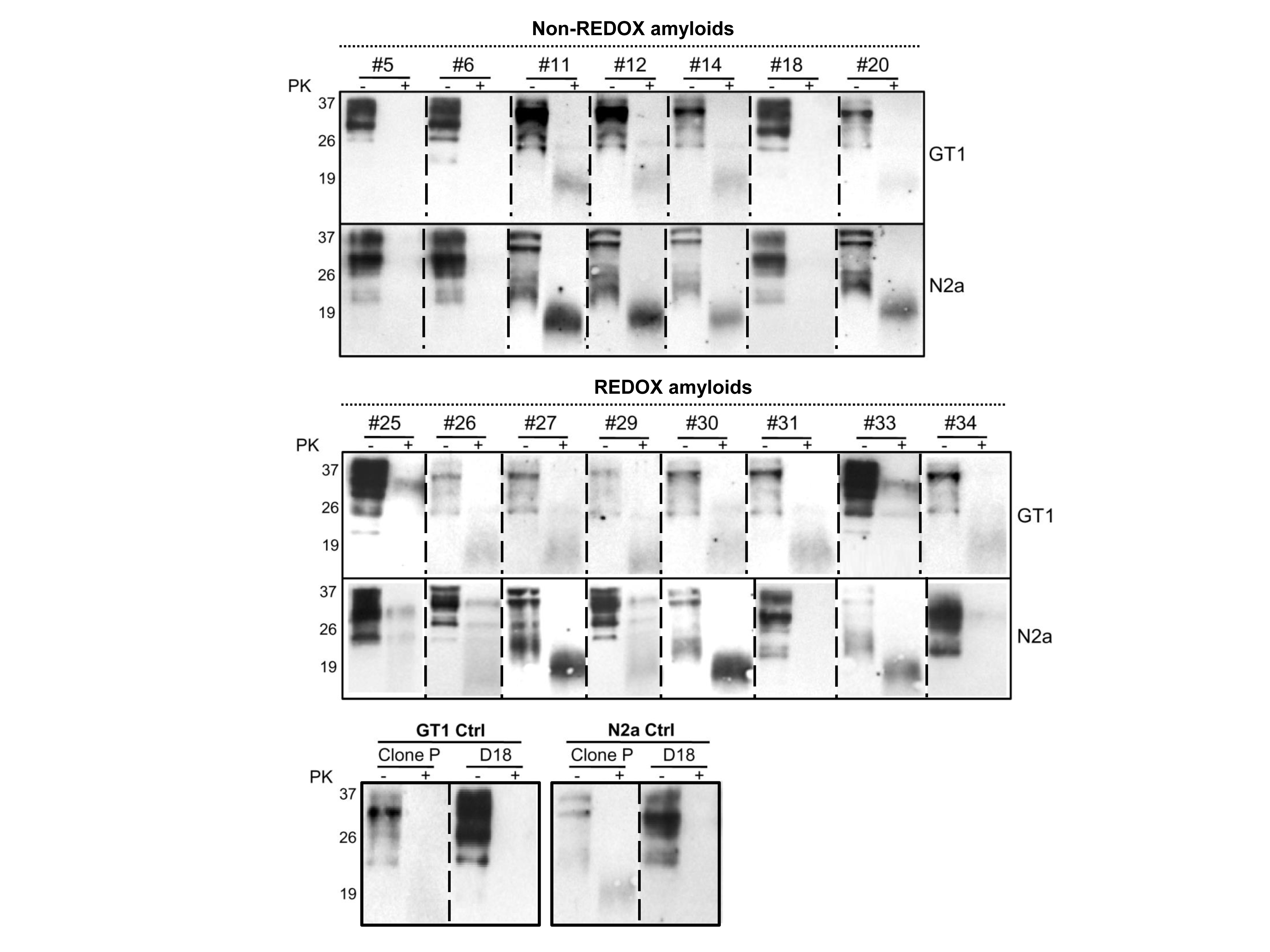

Supplement: S5 Fig — Seeding of recMoPrP(23–231) amyloid preparations induced the conversion of endogenous PrPC into mild protease K (PK) resistant forms. Western blotting shows the partial PK-resistance of neuroblastoma N2a and mouse hypothalamic GT1 amyloid fibril-infected cell lysates. Fibril-infected cell lysates (PK- lanes) were digested with PK at ratio 1:500 (w/w) (PK+ lanes). Western blots were performed using Fab D18 monoclonal antibody (1μg/mL) and Clone P (1μg/mL). Blots were developed with the enhanced chemiluminescent system (ECL, Amersham Biosciences) and visualized on Hyperfilm (Amersham Biosciences) (TIF) [file ppat.1005354.s008.tif]

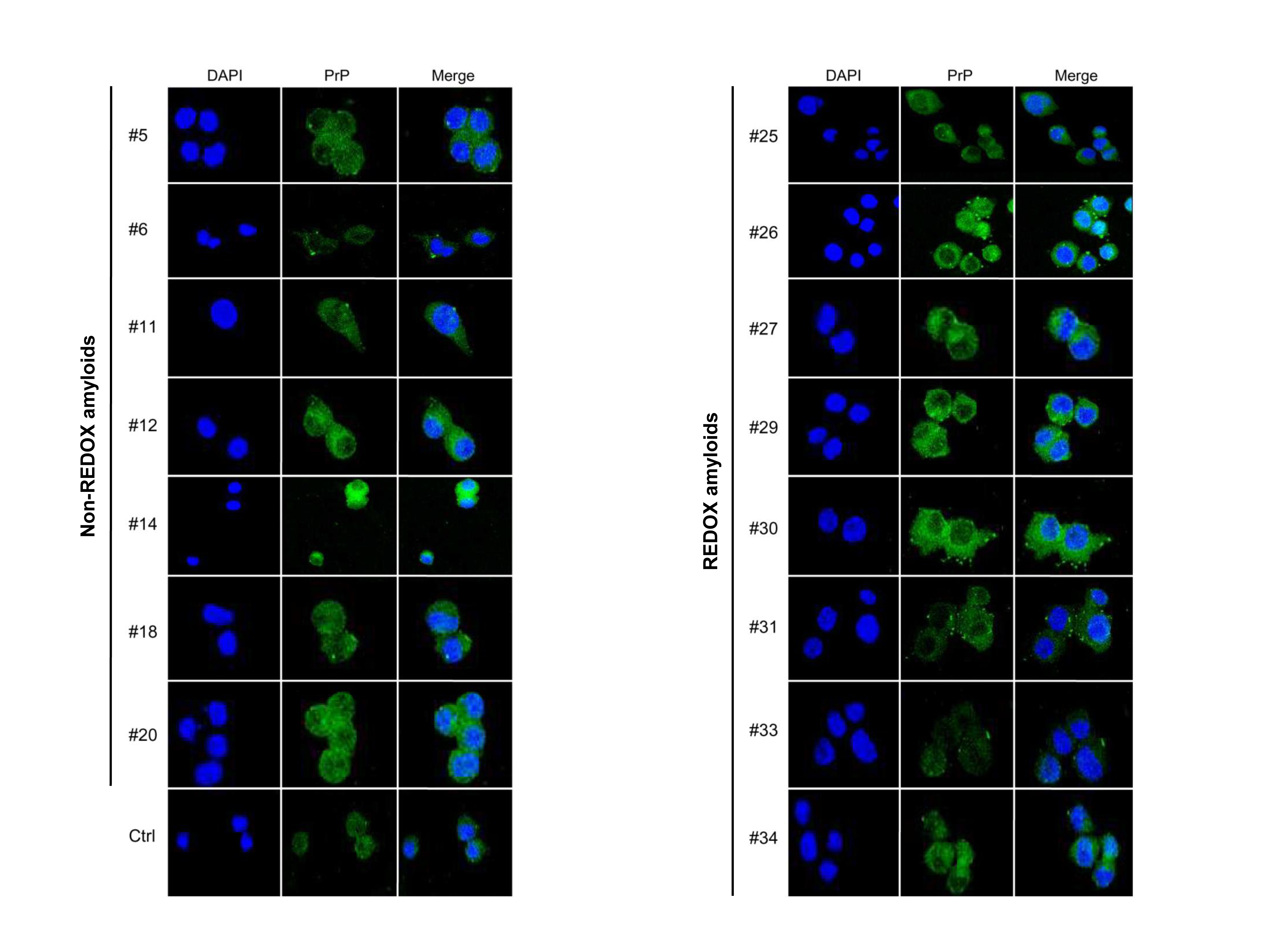

Supplement: S6 Fig — Accumulation of PrP was observed in neuroblastoma N2a cell lines infected with different amyloid preparations. The depositions and level of PrP (green) after six passages (P6) were detected by Fab D18 anti PrP antibody (10 μg/mL final concentration), using immunofluorescence. The nuclei (blue) were stained with DAPI. (TIF) [file ppat.1005354.s009.tif]

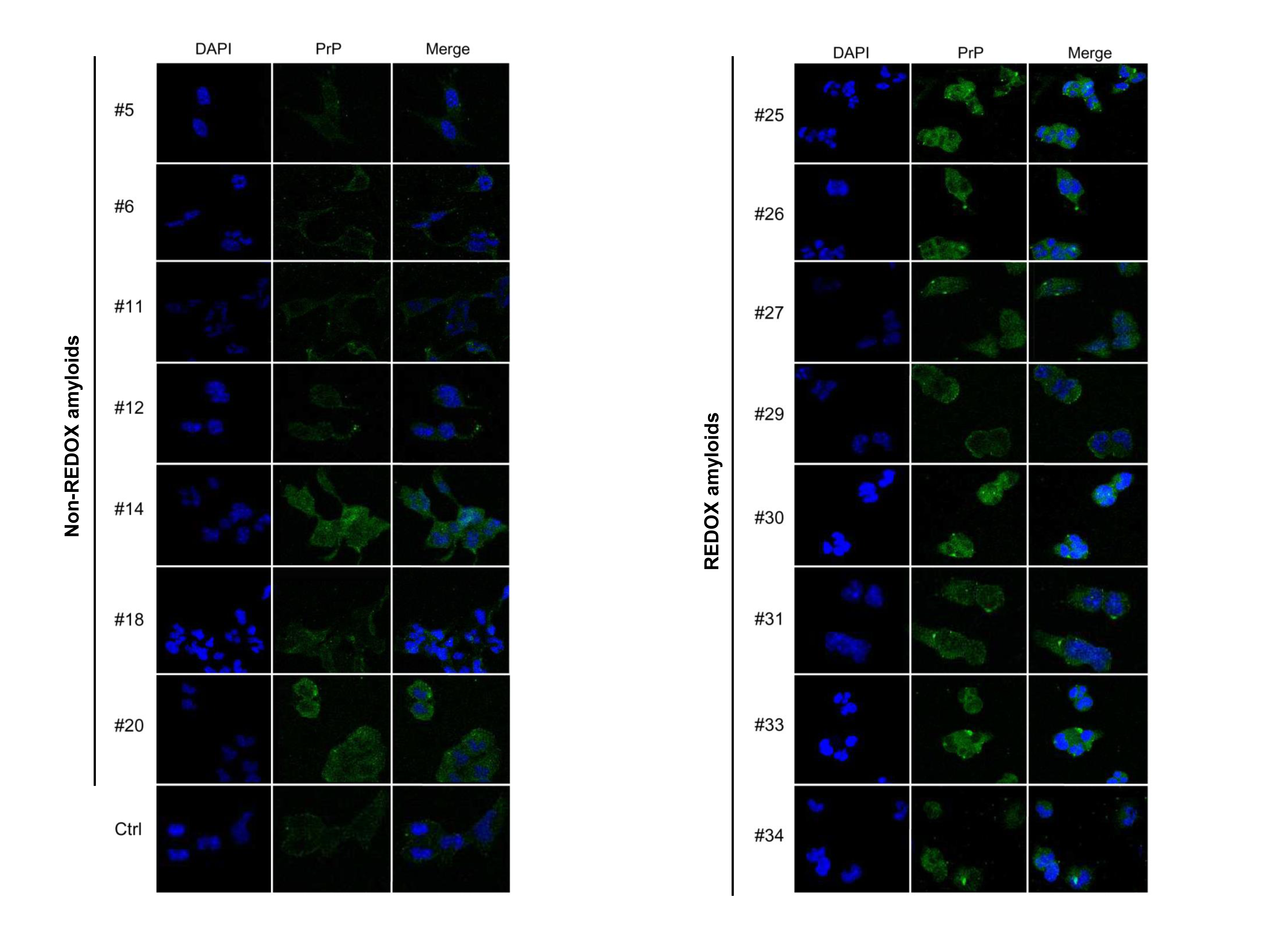

Supplement: S7 Fig — Accumulation of PrP was observed in mouse hypothalamic GT1 cell lines infected with different amyloid preparations. The depositions and level of PrP (green) after six passages (P6) were detected by Fab D18 anti PrP antibody (10 μg/mL final concentration), using immunofluorescence. The nuclei (blue) were stained with DAPI. (TIF) [file ppat.1005354.s010.tif]

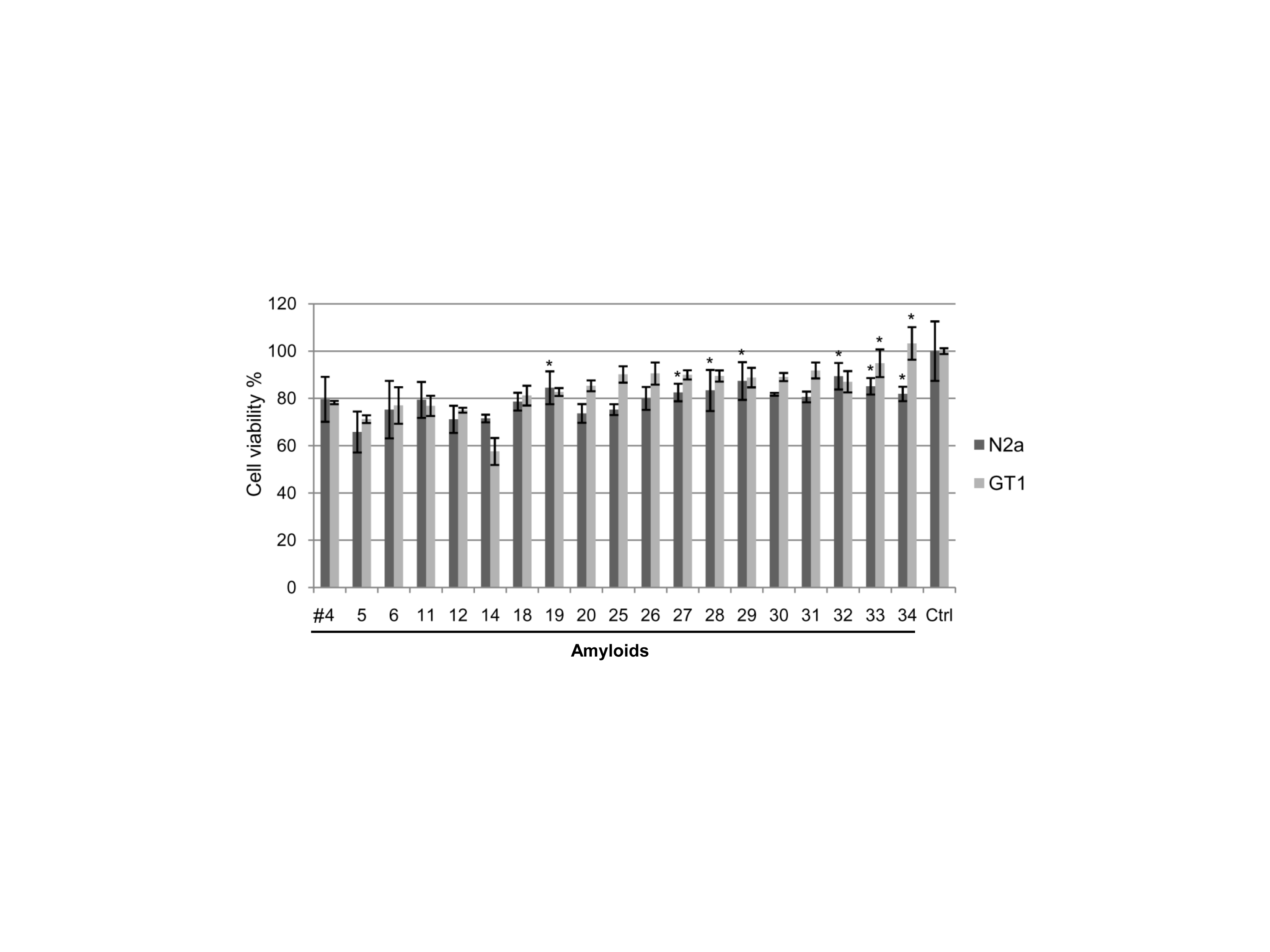

Supplement: S8 Fig — Amyloid fibrils of recMoPrP(23–231) showed mild toxicity in infected-cell cultures. Cell viability based on mitochondrial activity was measured by MTT assay. Control cells without treatment were counted as 100%. Bars indicate Standard deviation. t-test analysis were performed (*, P>0.05, n = 6). (TIF) [file ppat.1005354.s011.tif]

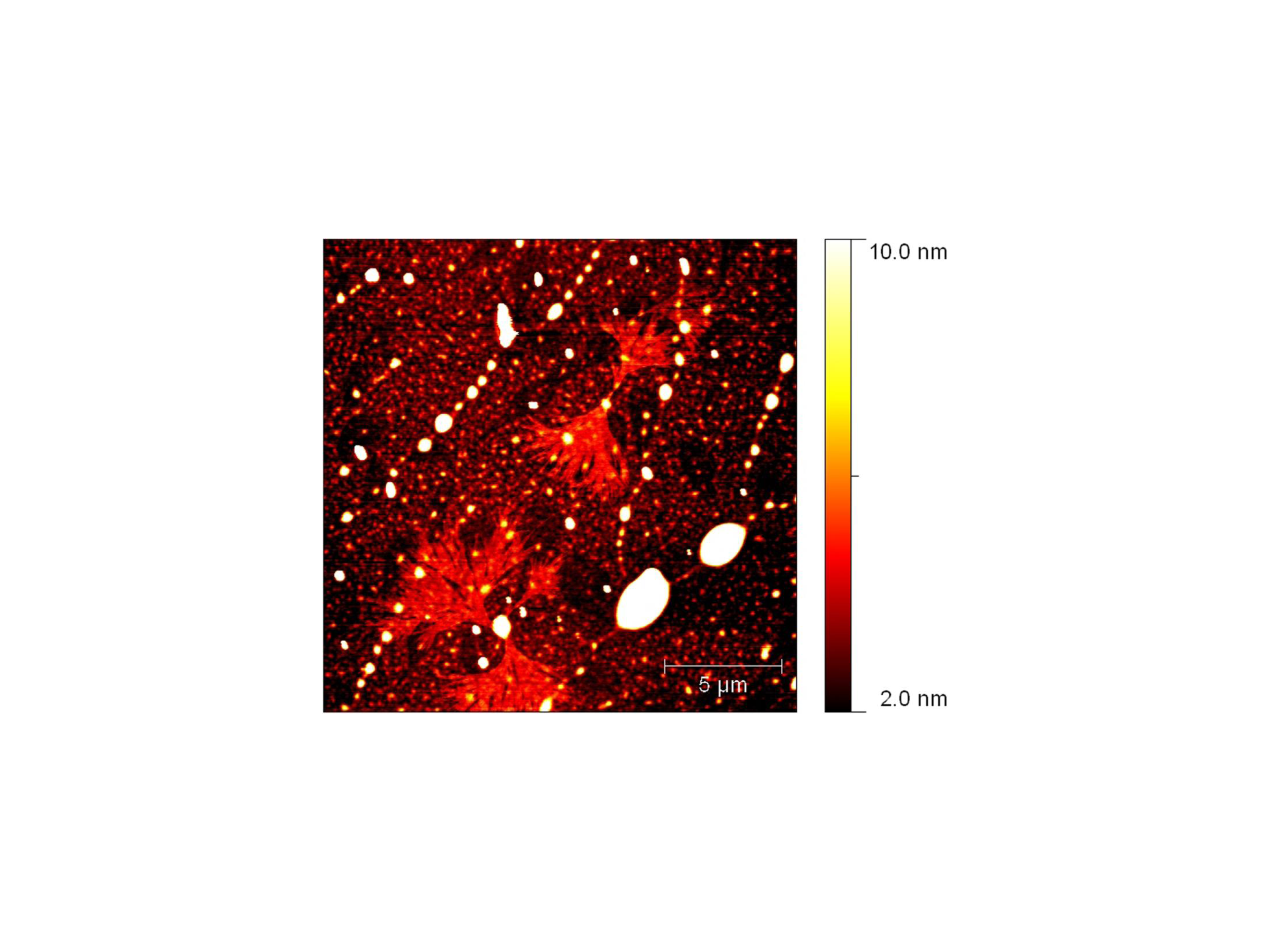

Supplement: S9 Fig — AFM imaging analyses were carried out at the end of the fibrillization reaction of amyloid preparation #18 (non-REDOX) after 72 hours with 15 minutes of interval-shaking time. AFM scan topographical image of PrP amyloid #18 deposited on mica surface showed classical forms of diffusion-limited aggregation. (TIF) [file ppat.1005354.s012.tif]

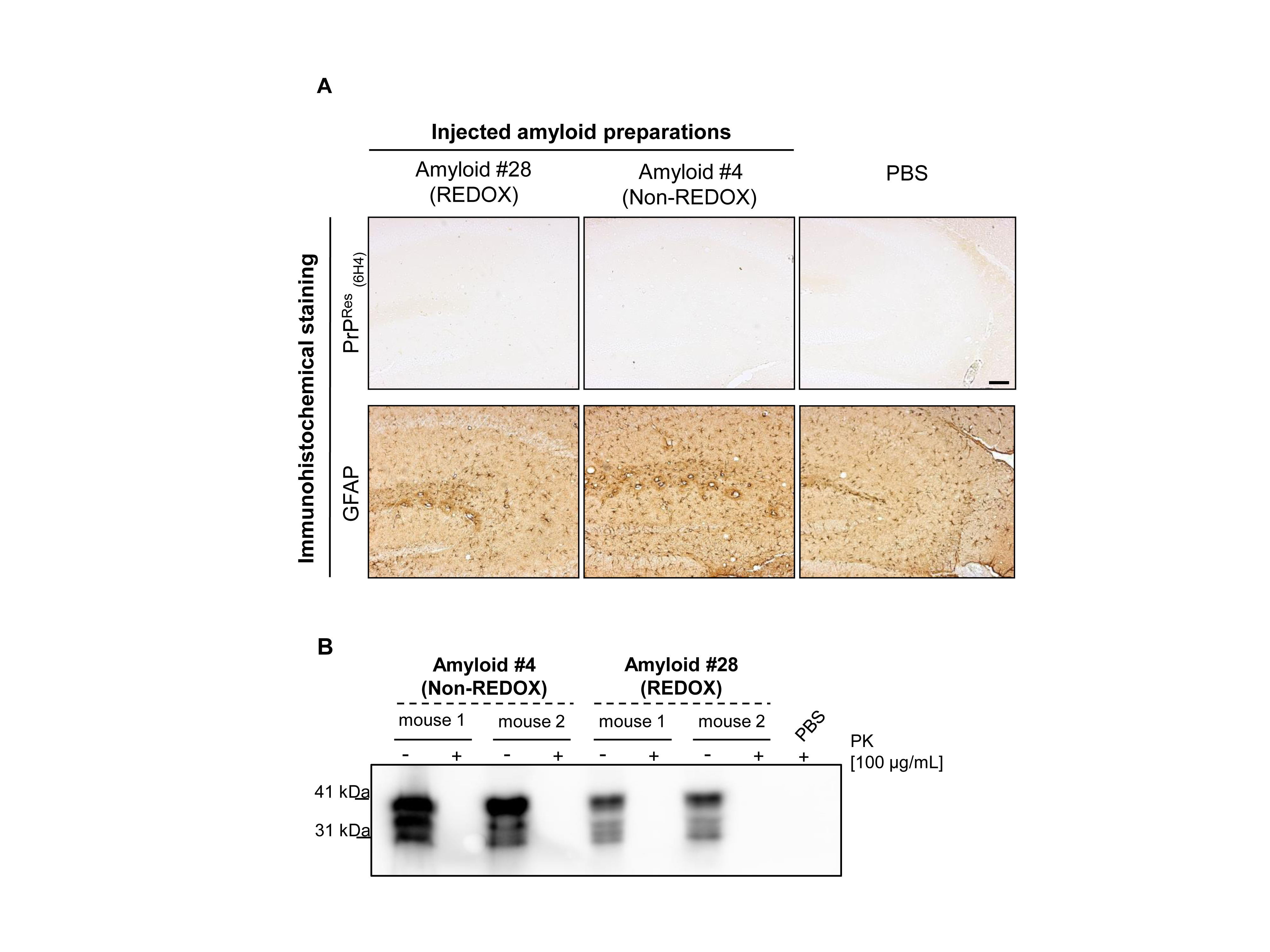

Supplement: S10 Fig — Neuropathological assessment of CD1 mice injected with preparation #4 or #28. Immunohistochemical analysis revealed the lack of PrPRes deposition in the brain associated to mild astroglial activation which is similar to that of the animal injected with PBS. Scale bar is 10 μm (A). Western blot analysis confirmed the lack of PrPRes deposition in the brain of injected animals. Samples were digested with PK (100 μg/mL) and immunoblotted with 6D11 monoclonal antibody to PrP (0.2 μg/mL, Covance) (B). Blots were developed with the enhanced chemiluminescent system (ECL, Amersham Biosciences) and visualized using a G:BOX Chemi Syngene system. (TIF) [file ppat.1005354.s013.tif]

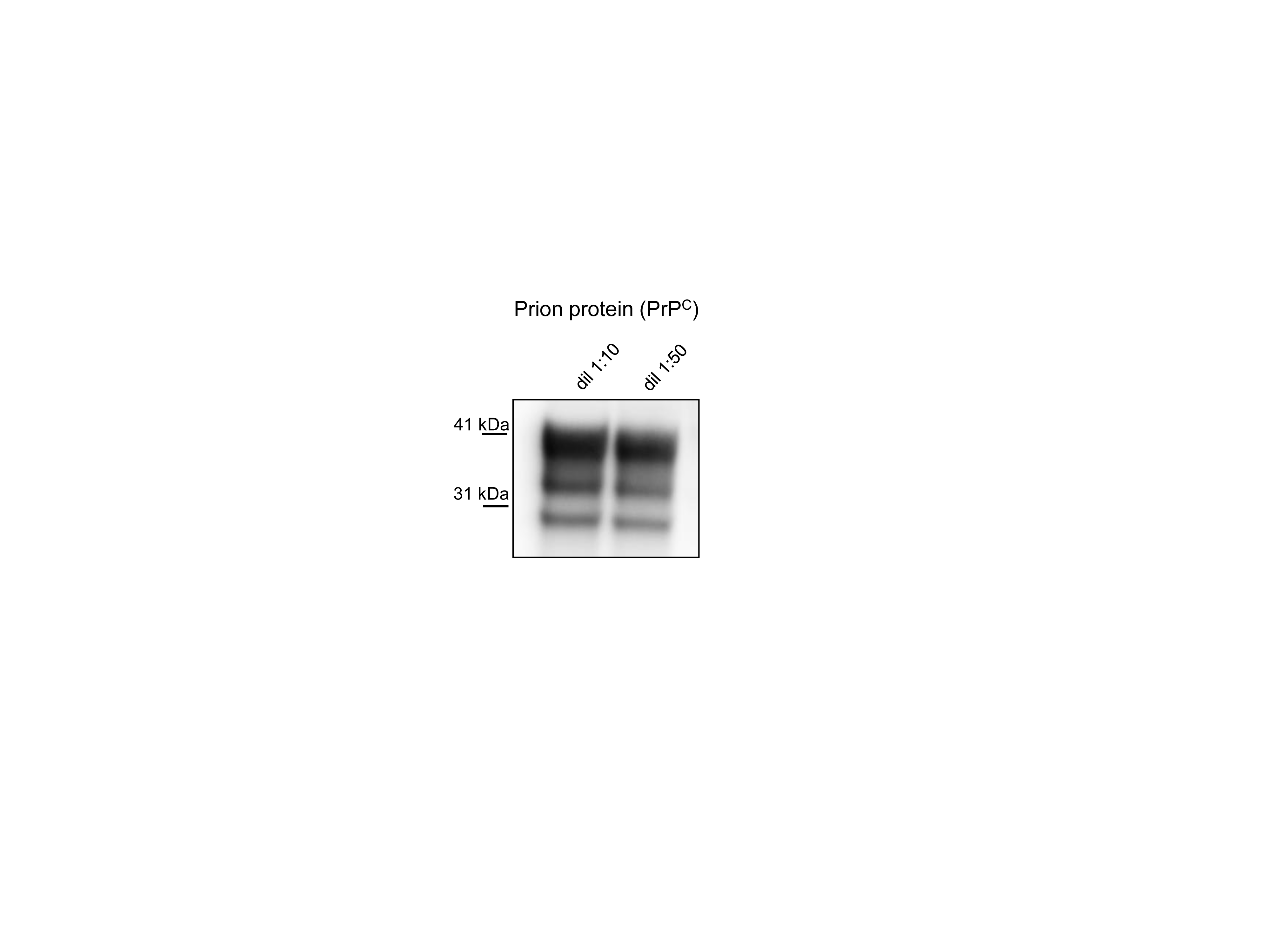

Supplement: S11 Fig — Western blot analysis of PrPC biochemical profile in the brain of CD1 mice (dilutions 1:10 and 1:50). Western blots were performed using 6D11 monoclonal antibody to PrP (0.2 μg/mL, Covance). Blots were developed with the enhanced chemiluminescent system (ECL, Amersham Biosciences) and visualized using a G:BOX Chemi Syngene system. (TIF) [file ppat.1005354.s014.tif]

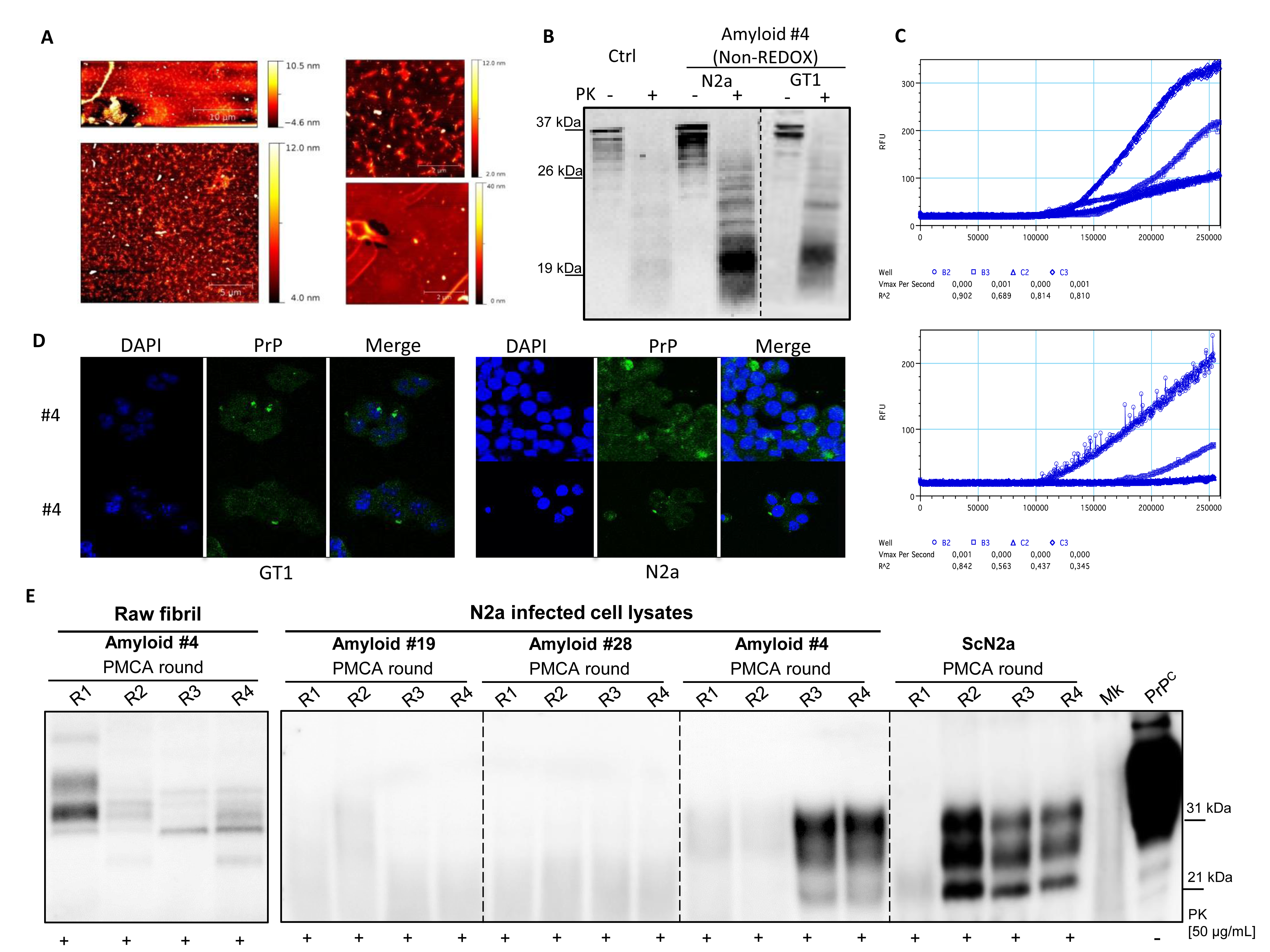

Supplement: S12 Fig — To assess whether the infectious amyloid #4 was obtained in a random phenomenon, we have used the same biochemical conditions to produce a new batch of this amyloid (#4) and we have assessed its infectious properties either in cells and by means of PMCA. Atomic Force Microscopy (AFM) imaging analysis of the new batch of amyloid #4 was performed at the end of the fibrillization reactions after 72 hours. AFM scan topographical images of prion protein (PrP) deposited on mica surface, large-scale images (A); Independent fibrillization kinetic experiments with ThT as fluorescence dye (B); Western blots of amyloid #4 infected cell lysates (N2a and GT1) after PK digestion (2μg/mL) incubated with the Clone P monoclonal antibody (1 μg/mL final concentration) (C); Immunofluorescence staining of amyloid #4-infected N2a and GT1 cells using anti-PrP monoclonal antibody Fab D18 (10 μg/mL final concentration) (D); Seeding ability of new batch of amyloid #4 (raw fibril) was assessed by means of PMCA using brain homogenates of CD1 mice as substrates for amplification. New batches of amyloid #4, #19 and #28 were inoculated in N2a cells, collected at P6 and amplified in PMCA. Amplification of ScN2a infected cell lysate was used as internal control for PMCA reaction. Western blots were performed using 6D11 monoclonal antibody to PrP (0.2 μg/mL, Covance). Blots were developed with the enhanced chemiluminescent system (ECL, Amersham Biosciences) and visualized using a G:BOX Chemi Syngene system (E). (TIF) [file ppat.1005354.s015.tif]
